# The predominant PAR4 variant in individuals of African ancestry worsens murine and human stroke outcomes

**DOI:** 10.1172/JCI169608

**Published:** 2023-09-15

**Authors:** Frederik Denorme, Nicole D. Armstrong, Michelle L. Stoller, Irina Portier, Emilia A. Tugolukova, Rikki M. Tanner, Emilie Montenont, Seema Bhatlekar, Mark Cody, John L. Rustad, Abigail Ajanel, Neal D. Tolley, Darian C. Murray, Julie L. Boyle, Marvin T. Nieman, Steven E. McKenzie, Christian Con Yost, Leslie A. Lange, Mary Cushman, Marguerite R. Irvin, Paul F. Bray, Robert A. Campbell

**Affiliations:** 1 Program in Molecular Medicine and; 2 Department of Neurology, Division of Vascular Neurology, University of Utah, Salt Lake City, Utah, USA.; 3 Department of Epidemiology, University of Alabama at Birmingham, Birmingham, Alabama, USA.; 4 Bioinformatics Shared Resource, Huntsman Cancer Institute, University of Utah, Salt Lake City, Utah, USA.; 5 Department of Pharmacology, Case Western Reserve University, Cleveland, Ohio, USA.; 6 Department of Medicine, The Cardeza Foundation for Hematologic Research, Thomas Jefferson University, Philadelphia, Pennsylvania, USA.; 7 Department of Pediatrics, Division of Neonatology, University of Utah, Salt Lake City, Utah, USA.; 8 Division of Biomedical Informatics and Personalized Medicine, Department of Medicine, University of Colorado Anschutz Medical Campus, Aurora, Colorado, USA.; 9 Department of Medicine, Larner College of Medicine at the University of Vermont, Burlington, Vermont, USA.; 10 Department of Internal Medicine, Division of Hematology and Hematologic Malignancies, and; 11 Department of Pathology, Division of Microbiology and Immunology, University of Utah, Salt Lake City, Utah, USA.

**Keywords:** Hematology, Neuroscience, Drug therapy, Platelets, Stroke

## Abstract

Protease-activated receptor 4 (PAR4) (gene *F2RL3*) harbors a functional dimorphism, rs773902 A/G (encoding Thr120/Ala120, respectively) and is associated with greater platelet aggregation. The A allele frequency is more common in Black individuals, and Black individuals have a higher incidence of ischemic stroke than White individuals. However, it is not known whether the A allele is responsible for worse stroke outcomes. To directly test the in vivo effect of this variant on stroke, we generated mice in which *F2rl3* was replaced by *F2RL3*, thereby expressing human PAR4 (hPAR4) with either Thr120 or Ala120. Compared with hPAR4 Ala120 mice, hPAR4 Thr120 mice had worse stroke outcomes, mediated in part by enhanced platelet activation and platelet-neutrophil interactions. Analyses of 7,620 Black subjects with 487 incident ischemic strokes demonstrated the AA genotype was a risk for incident ischemic stroke and worse functional outcomes. In humanized mice, ticagrelor with or without aspirin improved stroke outcomes in hPAR4 Ala120 mice, but not in hPAR4 Thr120 mice. P selectin blockade improved stroke outcomes and reduced platelet-neutrophil interactions in hPAR4 Thr120 mice. Our results may explain some of the racial disparity in stroke and support the need for studies of nonstandard antiplatelet therapies for patients expressing PAR4 Thr120.

## Graphical abstract

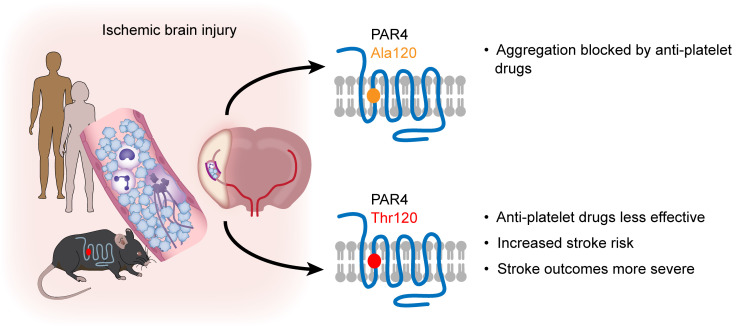

## Introduction

Stroke and ischemic heart disease are the most common causes of death in adults worldwide (1). Whereas myocardial infarction is primarily caused by coronary artery atherosclerosis, ischemic stroke (IS) has a more complex set of subtypes and underlying pathophysiologic mechanisms (2). Occlusive platelet thrombi contribute to cerebrovascular ischemia, and antiplatelet drugs are a first-line therapy for secondary prevention after IS or transient ischemic attack (TIA) (3).

Thrombin is a potent physiologic agonist mediating platelet activation, and platelet hyperreactivity to thrombin predicts acute ischemic events (4). Human platelets express 2 thrombin-activated GPCRs, protease-activated receptor 1 (PAR1) and PAR4. The PAR1 blocker vorapaxar is FDA approved for treatment of selected patients with ischemic coronary and peripheral vascular conditions, but is contraindicated in stroke (5). Compared with PAR1, PAR4 is less well studied, but recent findings emphasize its biologic importance and potential as a therapeutic target (6). PAR4 induces neutrophil rolling and adherence to the endothelium (7) and promotes platelet-neutrophil aggregate (PNA) formation (8). PNAs are elevated during IS and get trapped in the brain, leading to the release of neutrophil extracellular traps (NETs), which are neurotoxic and prothrombotic (9, 10).

Family and twin studies have repeatedly demonstrated genetic heritability as a major risk factor for IS (11). When grouped by race/ethnicity, Black individuals have a higher incidence of IS than White individuals, only some of which is explained by traditional risk factors (12–14). When grouped by geographic ancestry in genetic analyses, both heritability for platelet function and platelet activation through PAR4 are greater in Black than White subjects (15, 16). A common PAR4 Ala120Thr variant (SNP rs773902) contributes to this racial effect on PAR4-induced platelet aggregation (15, 16). Notably, the Thr120 allele frequency is approximately 0.615 in individuals of African ancestry and approximately 0.206 in individuals of European ancestry. To date, the evidence supporting hyperreactivity of PAR4 Thr120 compared with Ala120 is based on ex vivo studies in small numbers of healthy donors, but the nongenetic aspects of race/ethnicity and other confounding variables were not considered (15–19). Thus, direct experimental evidence for the Ala120Thr variant altering ex vivo or in vivo platelet function is lacking.

Compared with aspirin alone, dual antiplatelet therapy (DAPT) with aspirin and P2Y12 blockade using clopidogrel or ticagrelor may provide better stroke prevention, but generally has an increased risk of severe bleeding (20). It is noteworthy that despite DAPT, ex vivo platelet aggregation through PAR1 was normal in 31% and normal through PAR4 in 63% of patients (21). Such persistent platelet reactivity may limit effectiveness of DAPT; we believe it is unknown whether the clinical benefit of DAPT is modified by the PAR4 Ala120Thr variant.

The in vivo effects of the PAR4 Ala120Thr variant on either stroke or pharmacogenetic interactions with antiplatelet agents have not been studied. After using a transient middle cerebral artery occlusion (tMCAO) model of IS in our mice expressing human PAR4 (hPAR4), we report that compared with Ala120 mice, Thr120 mice have hyperreactive platelets and worse stroke outcomes. Unlike Ala120 mice, Thr120 mice derive no benefit from ticagrelor or DAPT, but do benefit from P selectin blockade. We also demonstrate the Thr120 variant is a stroke risk factor in a prospective cohort of self-identified Black individuals. Taken together, these findings suggest a therapeutic approach for improving stroke outcomes in individuals with the Thr120 variant.

## Results

### Generation of hPAR4 Ala120 and hPAR4 Thr120 mice.

**Figure 1:**
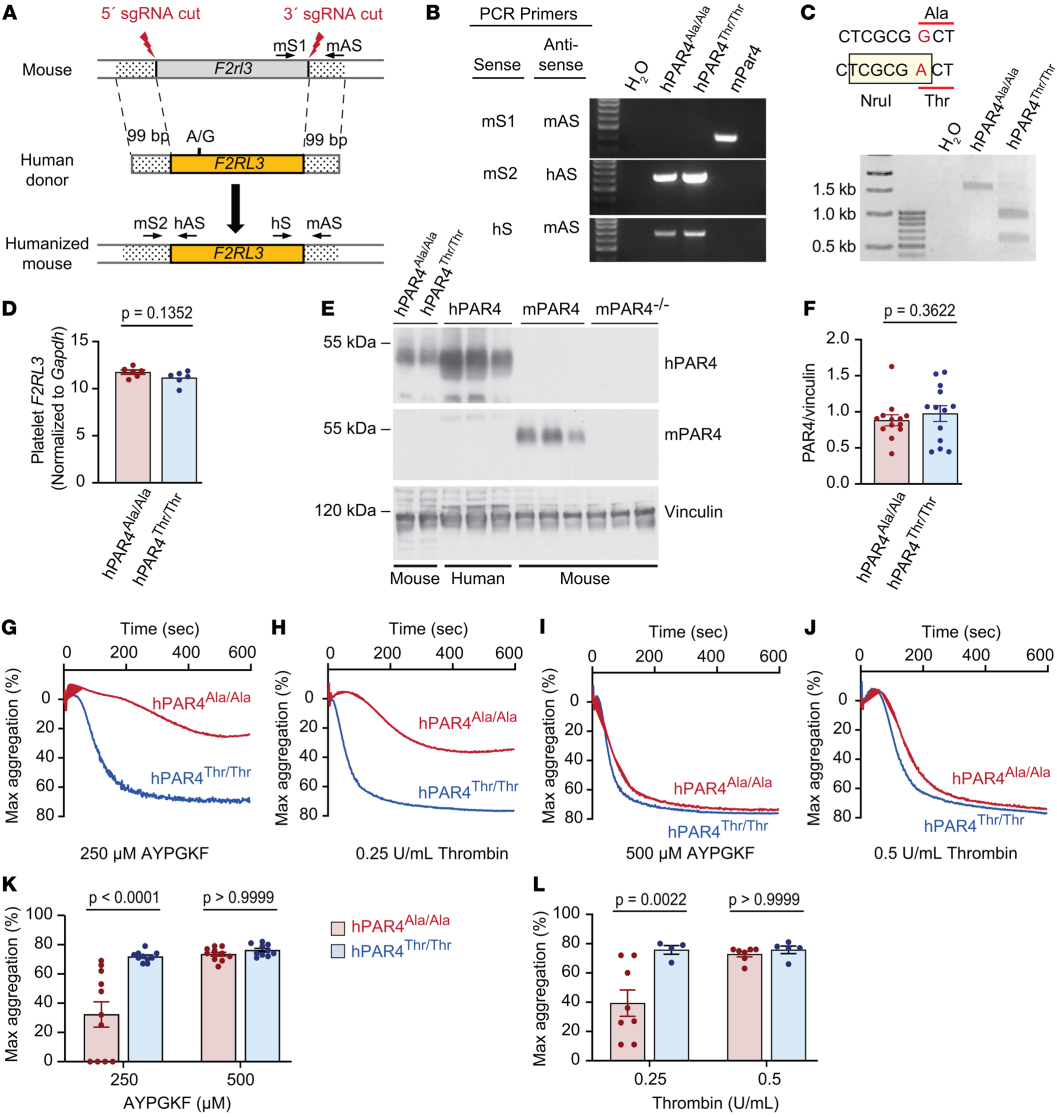
Characterization of hPAR4 mice. (**A**) Schematic showing generation of hPAR4 mice. PCR primers are shown for *F2rl3* (mouse sense 1 [mS1] and mouse antisense [mAS]), the 5′ insertion of *F2RL3* (mouse sense 2 [mS2] and human antisense [hAS]), and the 3′ insertion of *F2RL3* (hS and mAS). (**B**) Agarose gel of PCR products amplified from genomic DNA from hPAR4 and C57BL/6 (mPAR4) mice. Image is representative of 3 independent experiments. (**C**) PAR4 sequence with SNP for each allele in bold and codon in red underline. Boxed sequence shows a *Nrul* restriction site only in the *F2RL3* variant containing the A allele (hPAR4^Thr/Thr^). Agarose gel shows PCR-amplified genomic DNA digested with *NruI*. Image is representative of 3 independent experiments. (**D**) *F2RL3* expression was assessed by quantitative real-time PCR using mRNA isolated from platelets from hPAR4^Ala/Ala^ and hPAR4^Thr/Thr^ mice. Normality determined by Shapiro-Wilk test, with significance determined by unpaired *t* tests. *n* = 6 per group. (**E**) Platelets from C57BL/6 mice (mPAR4) and global PAR4-deficient mice (mPAR4^–/–^) were stained for human PAR4 (upper blot) or murine PAR4 (middle blot). Human platelets (hPAR4) were used as controls. (**F**) Platelet PAR4 protein levels in hPAR4^Ala/Ala^ and hPAR4^Thr/Thr^ mice (*n* = 13 per group). Normality determined by Shapiro-Wilk test with significance determined by Mann-Whitney *U* test. (**G**–**L**) Platelets were isolated from hPAR4^Ala/Ala^ and hPAR4^Thr/Thr^ mice and stimulated with either AYPGKF or human thrombin. Representative tracings for AYPGKF (*n* = 10–11 per group; **G** and **I**) and thrombin (*n* = 4–8 per group; **H** and **J**). (**K** and **L**) Maximal aggregation of AYPGKF (*n* = 10–11 per group) and thrombin (*n* = 4–8 per group). Significance was determined by 2-way ANOVA with Bonferroni’s multiple-comparison test (**K**) and a mixed-effects analysis with Bonferroni’s multiple-comparisons test (**L**).

We sought to dissect the direct role of rs773902 on platelet hyperactivity through the generation of humanized mice homozygous for the G allele (Ala120) or the rs773902 A allele (Thr120). hPAR4 Ala120 and Thr120 mice were generated using CRISPR/Cas9 (22) with sgRNAs against 5′ and 3′ *F2rl3* (mouse gene encoding Par4 protein) and with an *F2RL3* (human gene encoding PAR4 protein) donor strand and 99 bp homologous arms corresponding to the 5′ and 3′end of *F2rl3* (Figure 1A). This strategy allowed for the insertion of full-length human *F2RL3* cDNA under the control of mouse gene regulatory elements. The hPAR4 Ala120 line was generated on a C57BL/6 background first, with offspring from this line used to generate the hPAR4 Thr120 line (Figure 1A). Whole genome sequencing (WGS) confirmed *F2RL3* in the correct genomic site for both the hPAR4 Ala120 and Thr120 lines (Supplemental Figure 1; supplemental material available online with this article; https://doi.org/10.1172/JCI169608DS1). Both the hPAR4 Ala120 and hPAR4 Thr120 strains were backcrossed 8 times onto a C57BL/6J background (23). Heterozygous mice from both lines were bred together to generate homozygous hPAR4 Ala120 and hPAR4 Thr120 mice, designated hPAR4^Ala/Ala^ and hPAR4^Thr/Thr^, respectively.

Backcrossed hPAR4^Ala/Ala^ and hPAR4^Thr/Thr^ mice contained a 5′ mouse/human junction and a 3′ human/mouse junction (Figure 1, A and B). Neither strain contained a 3′ mouse/mouse junction that was observed in Par4 WT C57BL/6J mice (mPAR4, Figure 1B). Digestion of a PCR-amplified fragment of genomic DNA from hPAR4^Thr/Thr^ mice with Nrul confirmed the presence of the desired rs773902 A allele (Figure 1C). Assessment of expression in tissues known to express *F2RL3* (24) demonstrated similar levels of *F2RL3* mRNA in platelets, liver, brain, and lung between both lines (Figure 1D and Supplemental Figure 2). Figure 1E shows hPAR4 mouse platelets express the expected approximately 45 kD hPAR4 protein, whereas no expression was observed in platelets from WT C57BL/6J mice (mPAR4) or PAR4-deficient mice (mPar4 KO). Only WT C57BL/6J mice expressed murine PAR4 (Figure 1E). Importantly, platelet PAR4 expression was similar between hPAR4^Ala/Ala^ and hPAR4^Thr/Thr^ mice (Figure 1, E and F, and Supplemental Figure 3). Additionally, hPAR4 expression in humanized murine platelets appeared similar to murine Par4 expression in platelets isolated from WT mice (Figure 1E). Complete blood cell counts including platelet counts and mean platelet volume were similar between hPAR4^Ala/Ala^ and hPAR4^Thr/Thr^ mice (Supplemental Table 1). Platelet half-life and platelet production after depletion were similar between hPAR4^Ala/Ala^ and hPAR4^Thr/Thr^ mice (Supplemental Figure 4) and similar to what was shown in published reports using WT mice (25, 26). Platelet morphology, granule number, and granule content including fibrinogen and platelet factor 4 (PF4), were comparable between hPAR4^Ala/Ala^ and hPAR4^Thr/Thr^ mice (Supplemental Figure 5).

### Platelets from hPAR4^Thr/Thr^ mice are hyperreactive.

Compared with platelets from hPAR4^Ala/Ala^ mice, platelets from hPAR4^Thr/Thr^ mice demonstrated significantly greater aggregation in response to low concentrations of PAR4-specific activating peptide (AYPGKF) and thrombin (Figure 1, G, H, and K–L). This genotype effect on platelet aggregation was lost with higher concentrations of AYPGKF and thrombin (Figure 1, I–L). There was no difference in ex vivo platelet aggregation between hPAR4^Ala/Ala^ and hPAR4^Thr/Thr^ mice when platelets were activated with collagen (Supplemental Figure 6, A–C). Consistent with the collagen-dependent aggregation, P selectin expression after stimulation with convulxin (CVX), a GPVI agonist, was similar between hPAR4^Ala/Ala^ mice and PAR4^Thr/Thr^ mice (Supplemental Figure 6D). To assess whether the variant caused PAR4-dependent signaling differences, we examined AKT activation, a regulator of PAR4-dependent platelet activation (27), and observed a significant increase in phosphorylated AKT (pAKT) after AYPGKF activation of hPAR4^Thr/Thr^ platelets compared with hPAR4^Ala/Ala^ platelets (Supplemental Figure 7). Moreover, PAR4-mediated platelet αIIbb3 activation and α-granule release were significantly greater in hPAR4^Thr/Thr^ mice than hPAR4^Ala/Ala^ mice for both AYPGKF and thrombin (Supplemental Figures 8 and 9). Phosphatidylserine (PS) exposure on the platelet surface is a defining feature of procoagulant platelets (28). Compared with stimulated platelets from hPAR4^Ala/Ala^ mice, hPAR4^Thr/Thr^ mice generated significantly more procoagulant platelets (Supplemental Figure 9E and Supplemental Figure 10).

For studying the effect of the hPAR4 Ala120Thr variant in in vivo murine thrombosis models, it was important to know the responsiveness of our humanized mice to *murine* thrombin. Not only were platelets from both hPAR4^Thr/Thr^ mice and hPAR4^Ala/Ala^ mice responsive to murine thrombin, but consistent with the human thrombin data, an increased reactivity of hPAR4^Thr/Thr^ platelets was observed (Supplemental Figures 11 and 12). Platelets expressing murine PAR4 had increased reactivity to murine thrombin compared with platelets from hPAR4^Thr/Thr^ and hPAR4^Ala/Ala^ mice (Supplemental Figure 11). In vivo hemostasis was assessed by a tail-clip bleeding assay and showed no significant difference between hPAR4^Ala/Ala^ and hPAR4^Thr/Thr^ mice (Supplemental Figure 13). Our results demonstrate 2 hPAR4 mouse lines have been generated that express functional human PAR4, and the PAR4 Thr120 variant is hyperreactive compared with the Ala120 variant.

### hPAR4^Thr/Thr^ mice have worse stroke outcomes.

**Figure 2:**
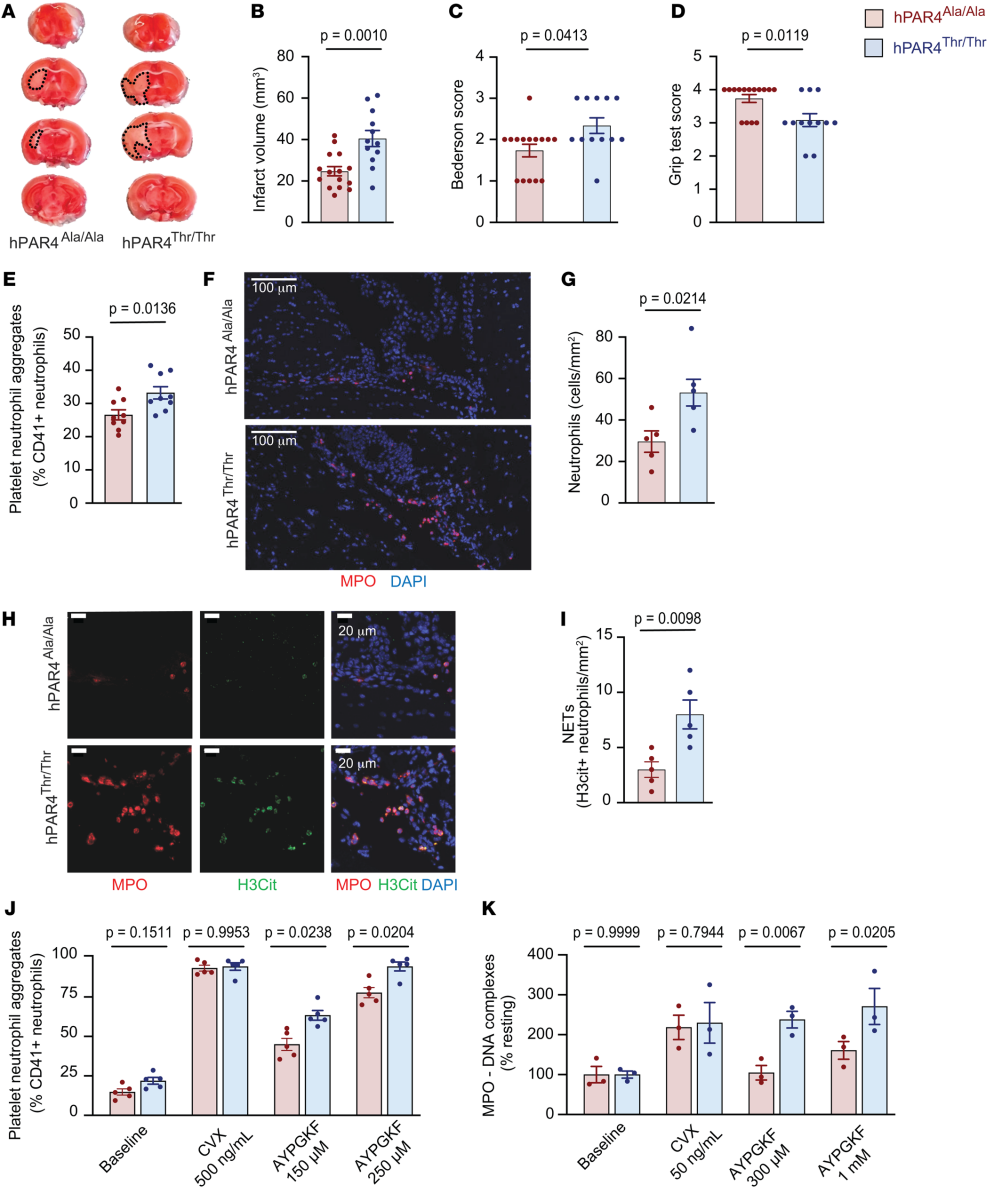
hPAR4^Thr/Thr^ mice have worse stroke outcomes than hPAR4^Ala/Ala^ mice in a moderate tMCAO model. Mice were subjected to 40 minutes of tMCAO followed by 23 hours of reperfusion (moderate). (**A**) Brain infarct volumes were quantified by staining with 2,3,5-triphenyl-tetrazolium chloride (TTC). Dead tissue is outlined with black dotted lines. Representative images from 12 to 15 mice per group. (**B**) Quantification of brain infarct volumes. Neurological and motor function were assessed by the Bederson (**C**) and grip (**D**) tests (10). Normality was determined by Shapiro-Wilk test, while significance was determined by an unpaired *t* test (**B**) and Mann-Whitney *U* test (**C** and **D**). *n* = 12–15 per group. (**E**) Average number of whole-blood PNAs based on Ly6G-CD41–positive events. *n* = 9 per group. (**F**) Representative images of neutrophils in infarcted brain tissue identified by myeloperoxidase (MPO) (red) and DNA (DAPI; blue) from *n* = 5 per group. (**G**) Number of neutrophils per infarcted brain area. *n* = 5 per group. (**H**) Representative images of NETs in brain tissue identified by MPO (red), citrullinated histone H3 (H3cit) (green), and DNA (DAPI; blue) from *n* = 5 per group. Images in **H** are magnified insets of images presented in **F**. (**I**) Percentages of NET-forming, H3cit-positive neutrophils. *n* = 5 per group. Normality was determined by Shapiro-Wilk test, while significance was determined by an unpaired *t* test (**G** and **I**). (**J**) Platelets and neutrophils were activated with AYPGKF, CVX, or vehicle (baseline). PNAs were measured by flow cytometry. Significance was determined by 2-way ANOVA with an uncorrected Fisher’s least significant difference (LSD). *n* = 5 per group. (**K**) Platelets and neutrophils from hPAR4^Ala/Ala^ and hPAR4^Thr/Thr^ mice activated with AYPGKF, CVX, or vehicle (baseline). NETs were quantified using an MPO-DNA ELISA. Significance was determined by 2-way ANOVA with Šidák’s multiple-comparisons test. *n* = 3 per group.

As platelet reactivity is known to regulate IS outcomes, we performed an IS model where brain ischemia was induced for 40 minutes by tMCAO, followed by 23 hours of reperfusion. A moderate stroke model (40 minutes ischemia) was initially selected because the PAR4 Ala120Thr variant difference in platelet aggregation was observed at submaximal stimulation of PAR4 (Figure 1, G and H). Twenty-four hours after initiation of artery occlusion (stroke onset), stroke outcomes were assessed and blood and brain tissue were analyzed. Compared with hPAR4^Ala/Ala^ mice, hPAR4^Thr/Thr^ mice had greater IS brain injury (Figure 2, A and B) and worse neurological and motor function (Figure 2, C and D).

When blood was analyzed 24 hours after stroke onset, circulating PNAs were mildly, but significantly, increased in hPAR4^Thr/Thr^ compared with hPAR4^Ala/Ala^ mice (Figure 2E and Supplemental Figure 14). Immunofluorescent analysis of ischemic brains showed that hPAR4^Thr/Thr^ mouse brains had increased infiltration of neutrophils (Figure 2, F and G) and NETs (Figure 2, H and I) compared with hPAR4^Ala/Ala^ mouse brains. We hypothesized that increased platelet-induced NET formation in hPAR4^Thr/Thr^ mice contributes to worsened IS outcomes and determined whether the PAR4 Thr120 variant affected ex vivo PNAs and platelet-induced NET formation. Figure 2J shows increased PNAs in hPAR4^Thr/Thr^ mice compared with hPAR4^Ala/Ala^ mice after stimulation of whole blood with AYPGKF, whereas no genotype difference was observed after GPVI stimulation. Furthermore, Figure 2K shows increased NET formation in response to incubation of neutrophils with AYPGKF-stimulated hPAR4^Thr/Thr^ platelets. To demonstrate the specificity for platelet PAR4 in NET induction, platelets and neutrophils were isolated from murine PAR4 WT mice and PAR4 KO mice. AYPGKF activated Par4 WT platelets, but not Par4 KO platelets, and induced NET formation using WT or PAR4 KO neutrophils (Supplemental Figure 15). In addition, AYPGKF failed to induce NET formation in WT or PAR4 KO neutrophils in the absence of WT platelets (Supplemental Figure 15). Taken together, these data indicate PAR4-dependent platelet activation and not PAR4-dependent neutrophil activation was responsible for the observed difference in NET formation.

**Figure 3:**
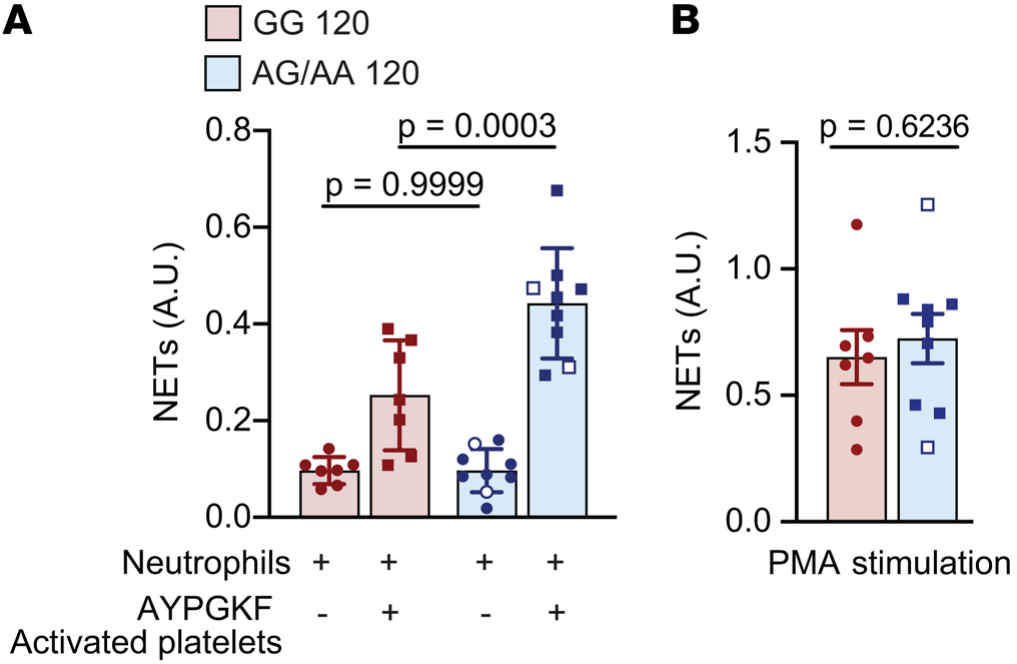
rs773902 AG/AA human platelets induce greater NET formation. (**A**) Platelets and neutrophils were isolated from donors of known rs773902 genotypes (AA, AG, and GG). Platelets were stimulated with AYPGKF or vehicle for 2.5 hours in the presence of neutrophils, and NETs were quantified using an MPO-DNA ELISA. *n* = 7–9 per group. Blue open symbols indicate AA genotype, while closed symbols indicate AT genotype. Significance was determined by 2-way ANOVA with Šidák’s multiple-comparisons test. (**B**) Neutrophils from the indicated groups were isolated and stimulated with PMA for 2.5 hours, and NETs were quantified using an MPO-DNA ELISA. *n* = 8 per group. Blue open symbols indicate AA genotype, while closed symbols indicate AT genotype. Normality was determined by Shapiro-Wilk test, while significance was determined by an unpaired *t* test.

To determine whether these findings apply to *human* platelets and neutrophils, we recruited 16 donors of a known rs773903 genotype and analyzed platelet-induced NET formation ex vivo in response to AYPGKF. As with the murine data, more NETs were induced by platelets from Thr120-positive donors (rs773902 AA+GA) than platelets from Thr-negative (rs773902 GG) donors (Figure 3). Notably, in the absence of platelets, phorbol myristate acetate–induced (PMA-induced) NET formation was not significantly different across genotypes, suggesting neutrophils from both genotypes have a similar potential to form NETs (Figure 3).

### The rs773902 genotype predicts stroke risk and neurologic outcome after human stroke.

We next assessed whether our murine findings translated to human IS genetics. Using a candidate gene approach, we analyzed a cohort of 7,620 self-identified Black individuals from the Reasons for Geographic and Racial Differences in Stroke (REGARDS) study who were free of baseline stroke, followed for an average of 10.2 years, with 487 experiencing an incident IS (Supplemental Figure 16). Supplemental Table 2 shows the baseline characteristics of the participants. Supplemental Table 3 shows antiplatelet drug use distribution at time of enrollment by rs773902 genotype. Supplemental Table 4 shows the genotype distribution among cases by IS subtype. As shown in Table 1, subjects with the rs773902 AA genotype had an HR for IS of 1.27 (CI 1.06–1.54, *P* = 0.012; base model) and fully adjusted HR of 1.26 (CI 1.04–1.53, *P* = 0.016). A similar association was observed when excluding cardioembolic (CE) stroke.

Table 2 shows that, among stroke cases, the rs773902 AA genotype was associated with unfavorable stroke outcome (modified Rankin score [mRS] 3–6). The signal was greater when CE stroke was excluded (2.13 OR; CI 1.16–3.92, *P* = 0.015, base model; and 2.04 OR; CI 1.10–3.79, *P* = 0.025, fully adjusted model 2). When unfavorable outcomes were further broken down by IS subtype, the rs773902 AA genotype had an increased risk for unfavorable outcome in the small vessel occlusion (SVO) subtype, although the number of patients in each group was small. We further modeled incident stroke and stroke outcomes using an additive and dominant model, as the effect of the allele for rs773902 is unknown in any human disease. Supplemental Tables 5 and 6 show similar trends for the effect of rs773902 in an additive model, but no effect in a dominant model.

### Direct blockade of human PAR4 improves murine stroke outcomes in both genotypes.

**Figure 4:**
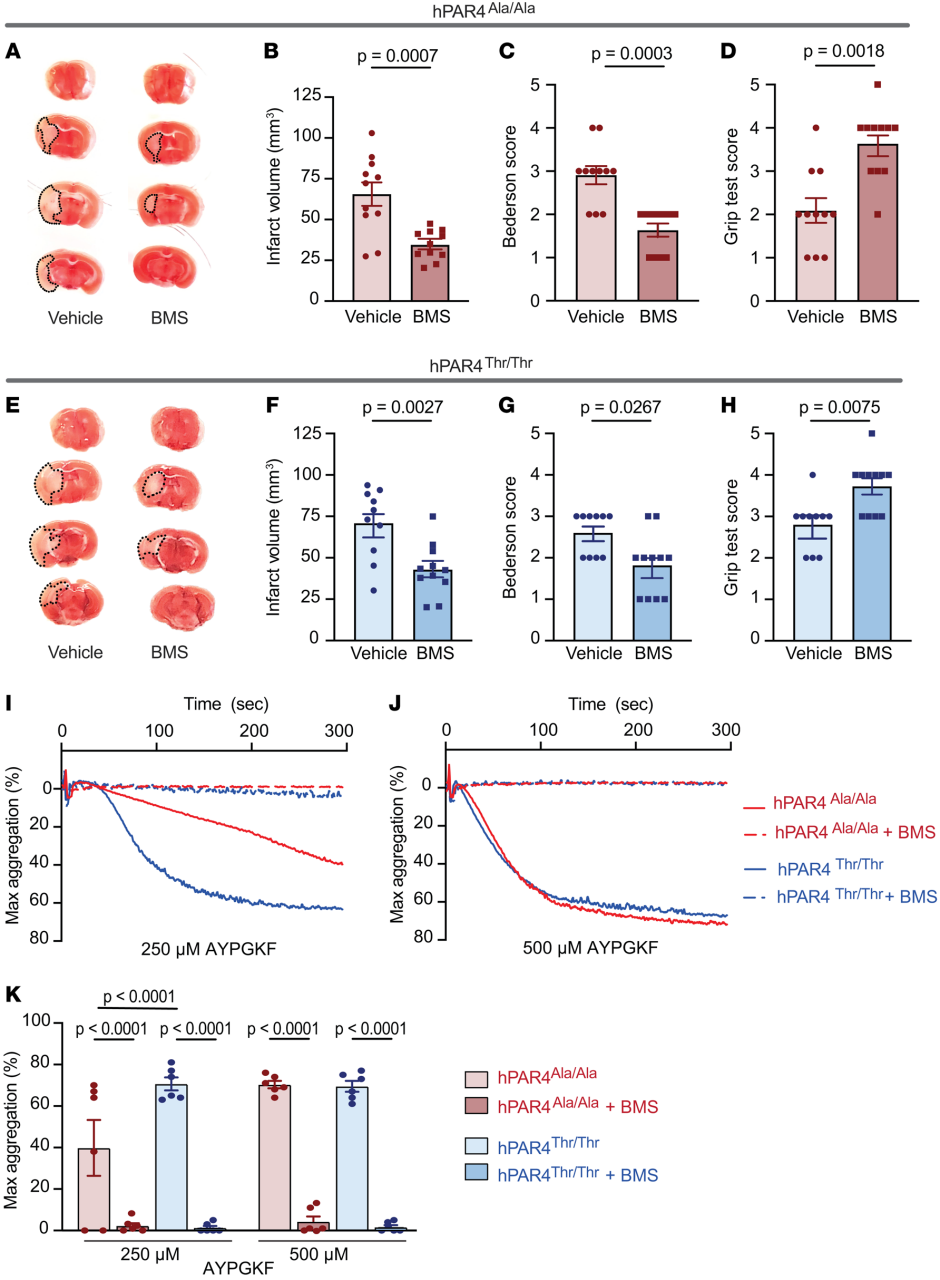
BMS-986120 attenuates IS brain injury in both hPAR4^Ala/Ala^ and PAR4^Thr/Thr^ mice. (**A**–**H**) BMS-986120 (10 mg/kg) was administered i.v. to hPAR4^Ala/Ala^ and hPAR4^Thr/Thr^ mice 1 hour before tMCAO. Mice were then subjected to 60 minutes of tMCAO (severe). (**A** and **E**) Brain tissue was stained with TTC. Dead brain tissue is outlined with black dotted lines. Representative images from *n* = 10–11. (**B** and **F**) Quantification of brain infarct volumes. Neurological and motor function were assessed by the Bederson (**C** and **G**) and grip (**D** and **H**) tests, respectively. Normality was determined by Shapiro-Wilk test, while significance was determined by an unpaired *t* test (**B** and **F**) and Mann-Whitney *U* test (**C**, **G**, **D**, and **H**). *n* = 10–11 per group. (**I**–**K**) BMS-986120 (10 mg/kg) was administered i.v. 1 hour before platelets were isolated from hPAR4^Ala/Ala^ and hPAR4^Thr/Thr^ mice. Washed platelets were resuspended in Tyrode’s buffer, and aggregation in response to AYPGKF was measured. Representative aggregation tracings in response to 250 μM (**I**) and 500 μM (**J**) AYPGKF are shown from *n* = 6 per group. Quantification of all curves is shown in **K**. Significance was determined by a 2-way ANOVA with Bonferroni’s multiple-comparisons test. *n* = 6 per group.

No human pharmacogenetics studies exist for rs773902 genotypes; therefore, we used our mouse strains as a preclinical model to assess rs773902 genotype effects on IS outcomes after antiplatelet therapies. For these studies, a longer occlusion time in the tMCAO model was used, since the infarct size in the hPAR4^Ala/Ala^ mice (Figure 2A) was too small to generate statistically appropriate comparisons with the hPAR4^Thr/Thr^ mice after treatment with antiplatelet agents. Notably, 60-minute occlusion has often been used in murine stroke studies (10, 26, 29–31). When mice were subjected to this more severe model of IS, the genotype difference in stroke outcomes and circulating PNAs was lost (Supplemental Figure 17). This is likely related to the loss of the genotype effect on aggregation with more intense platelet stimulation (Figure 1). Under these conditions, when hPAR4^Ala/Ala^ mice were pretreated with the preclinical PAR4-specific antagonist BMS-986120, an approximately 50% reduction in IS brain infarct volume was observed compared with in vehicle-treated mice (Figure 4, A and B). Neurological and motor function were also significantly ameliorated by BMS-986120 treatment (Figure 4, C and D). Similarly, in hPAR4^Thr/Thr^ mice, BMS-986120 treatment reduced infarct size by approximately 40% (Figure 4, E and F) and improved neurological and motor function (Figure 4, G and H). These experiments demonstrate stroke dependence on PAR4 in this murine model.

In parallel, we isolated platelets from BMS-986120–treated hPAR4^Ala/Ala^ and hPAR4^Thr/Thr^ mice and observed loss of AYPGKF-induced platelet aggregation and activation in both genotypes (Figure 4, I–K, and Supplemental Figure 18), while GPVI-dependent platelet aggregation remained unaffected (Supplemental Figure 19).

### P2Y12 blockade improves stroke outcomes in hPAR4^Ala/Ala^ mice, but not in hPAR4^Thr/Thr^ mice.

**Figure 5:**
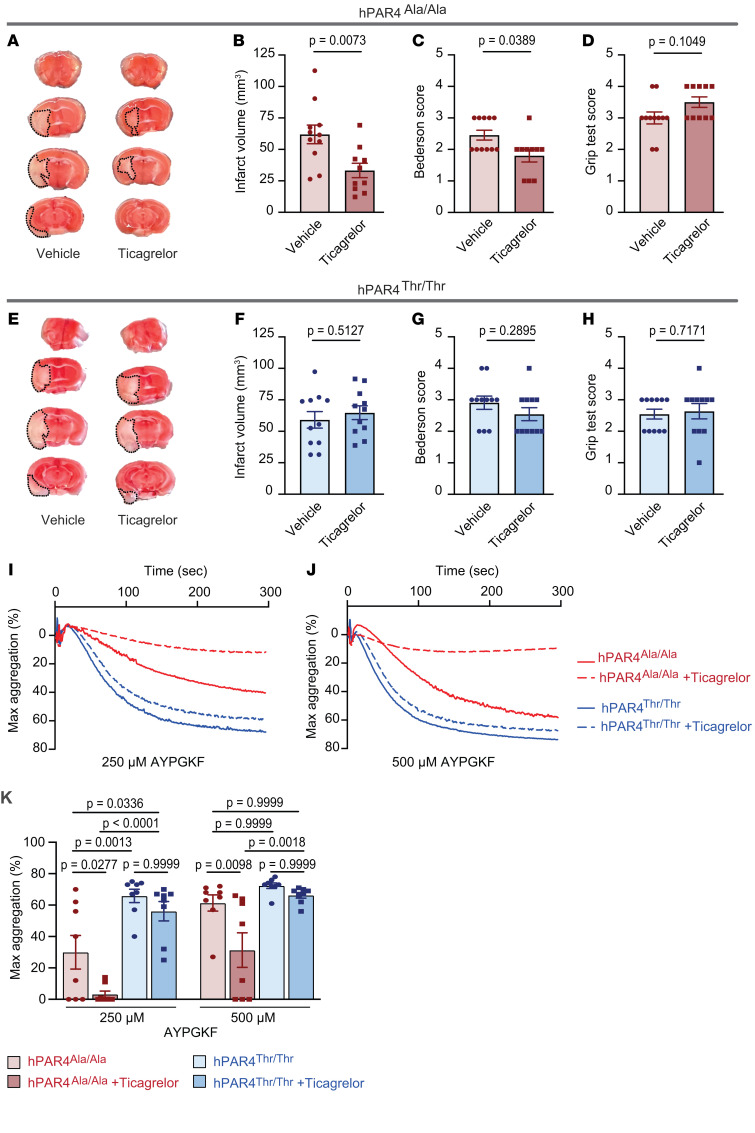
Ticagrelor attenuates IS brain injury only in hPAR4^Ala/Ala^ mice. Ticagrelor (200 mg/kg) or vehicle was administered by oral gavage to hPAR4^Ala/Ala^ and hPAR4^Thr/Thr^ mice 2 hours before tMCAO. Mice were then subjected to 60 minutes of tMCAO (severe). (**A** and **E**) Brain infarct volumes were quantified by staining with TTC, which colors live brain tissue red, while dead brain tissue remains white (outlined with black dotted lines). Representative images from *n* = 10–11 per group. (**B** and **F**) Quantification of brain infarct volumes. Neurological and motor function were assessed by Bederson (**C** and **G**) and grip (**D** and **H**) tests. Normality was determined by Shapiro-Wilk test, while significance was determined by an unpaired *t* test (**B** and **F**) and Mann-Whitney *U* test (**C**, **G**, **D**, and **H**). *n* = 10–11 per group. (**I**–**K**) Ticagrelor (200 mg/kg) was administered by gavage 2 hours before platelets were isolated from hPAR4^Ala/Ala^ and hPAR4^Thr/Thr^ mice. Washed platelets were resuspended in Tyrode’s buffer, and aggregation in response to AYPGKF was measured. Representative aggregation tracings in response to 250 μM (**I**) and 500 μM (**J**) AYPGKF are shown from *n* = 8 per group. Quantification of all curves is shown in **K**. Significance was determined by 2-way ANOVA with Bonferroni’s multiple-comparisons test.

P2Y12 blockade is a standard antiplatelet therapeutic approach used to prevent recurrent stroke. hPAR4^Ala/Ala^ and hPAR4^Thr/Thr^ mice were pretreated with the P2Y12 antagonist ticagrelor 2 hours before stroke onset, and outcomes were analyzed 24 hours later. P2Y12 blockade significantly protected hPAR4^Ala/Ala^ mice from IS brain injury compared with what occurred in vehicle-treated animals (Figure 5, A and B). In line with this, neurological behavior was significantly improved (Figure 5C) and motor function trended toward improvement (Figure 5D). In contrast, P2Y12 blockade did not affect stroke outcomes in hPAR4^Thr/Thr^ mice (Figure 5, E–H).

In hPAR4^Ala/Ala^ mice, ticagrelor blunted platelet aggregation in response to a low concentration of AYPGKF and reduced aggregation by 50% in response to a high AYPGKF concentration compared with platelets isolated from vehicle-treated animals (Figure 5, I–K). Analogous to the stroke results, P2Y12 blockade did not affect AYPGKF-induced platelet aggregation in hPAR4^Thr/Thr^ mice (Figure 5, I–K). Supplemental Figure 20 shows, importantly, that P2Y12-mediated platelet aggregation was equally blocked in hPAR4^Ala/Ala^ and hPAR4^Thr/Thr^ mice using this treatment regimen. This implies ticagrelor-mediated suppression of PAR4 signaling is required for its protective effect in murine stroke. Moreover, increasing the concentration of ticagrelor 2-fold to 400 mg/kg did not change IS outcomes in hPAR4^Thr/Thr^ mice compared with vehicle-treated mice (Supplemental Figure 21).

### Sustained thromboinflammation despite P2Y12 blockade in hPAR4^Thr/Thr^ mice.

**Figure 6:**
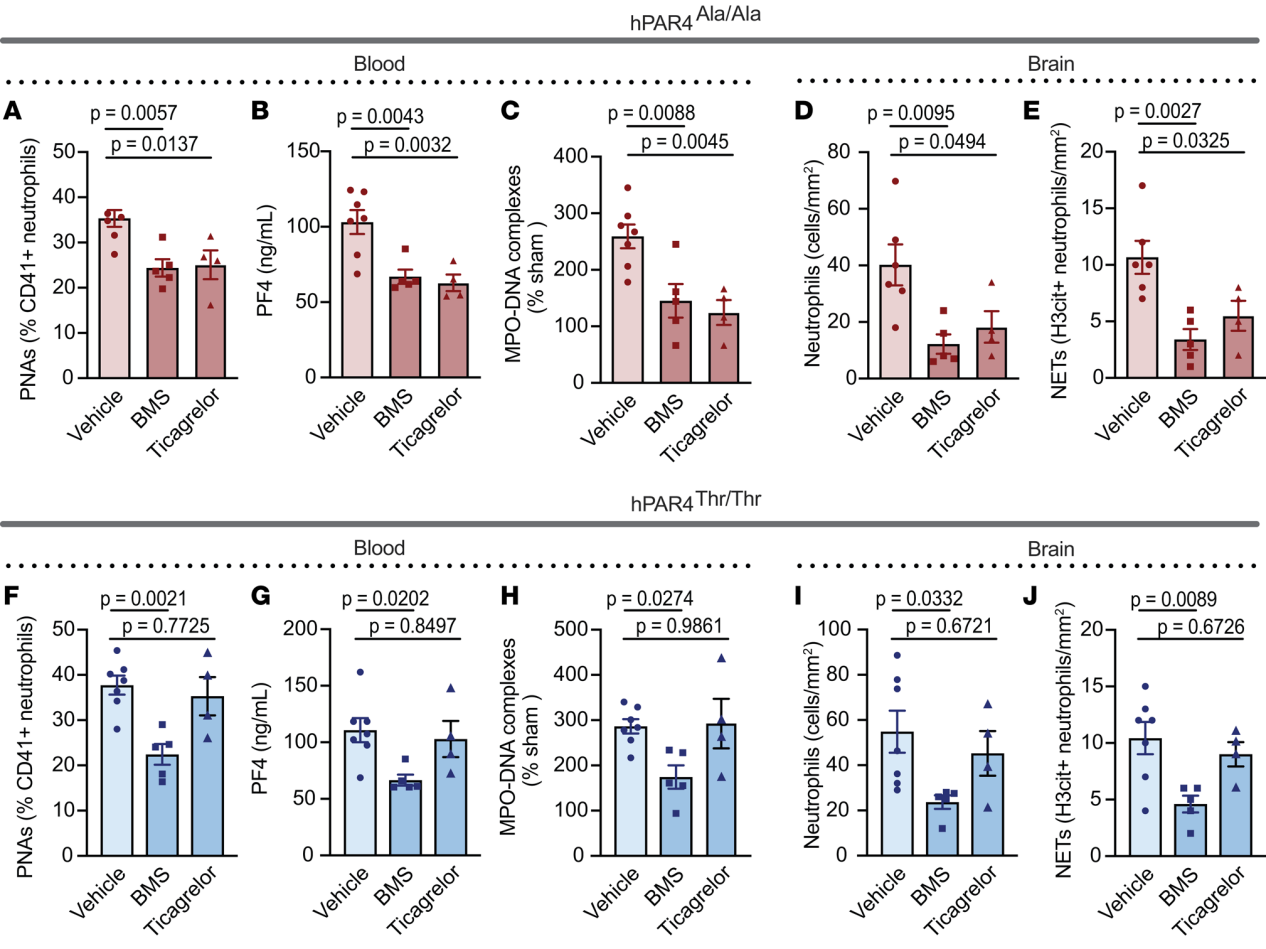
Potential mechanisms for BMS-986120 and ticagrelor protection after IS injury in hPAR4 mice. BMS-986120 (10 mg/kg) was administered i.v. 1 hour before tMCAO. Ticagrelor (200 mg/kg) was administered by oral gavage 2 hours before tMCAO. hPAR4^Ala/Ala^ and hPAR4^Thr/Thr^ mice were then subjected to 60 minutes of tMCAO followed by 23 hours of reperfusion (severe). Whole blood, plasma, and brains were isolated 24 hours after stroke. (**A** and **F**) PNAs were measured by flow cytometry by gating on Ly6G^+^ neutrophils that were positive for CD41^+^ platelets. *n* = 4–7 per group. (**B** and **G**) Platelet activation as assessed by PF4 release was measured in plasma by ELISA. *n* = 4–7. (**C** and **H**) NET formation in the plasma was measured using an MPO-DNA ELISA. *n* = 4–7 per group. (**D** and **I**) Neutrophils were identified by MPO and DNA (DAPI) statins and presented as neutrophils per area of infarcted brain tissue. *n* = 4–7 per group. (**E** and **J**) Brain tissue was stained for MPO, H3cit, and DNA (DAPI), and NET-forming neutrophils were quantified as the percentage of MPO-positive cells that were also H3cit positive. *n* = 4–7 per group. Normality was determined by Shapiro-Wilk test, while significance was determined by 1-way ANOVA with Dunnett’s multiple-comparisons test.

To explore mechanistic drivers of IS brain injury, thromboinflammation markers were measured in the circulation and brain tissue after stroke in both mouse strains treated in vivo with vehicle, BMS-986120, or ticagrelor. In hPAR4^Ala/Ala^ mice, both BMS-986120 and ticagrelor blocked PNAs, activated platelets and NET formation in the peripheral circulation after stroke compared with what occurred in vehicle-treated animals (Figure 6, A–C). Furthermore, examination of brains in hPAR4^Ala/Ala^ mice showed reduced neutrophils and NETs by pretreatment with either BMS-986120 or ticagrelor compared with vehicle (Figure 6, D and E). In hPAR4^Thr/Thr^ mice, BMS-986120 also lowered thromboinflammation markers in blood and brain tissue (Figure 6, F–J). However, ticagrelor did not reduce markers of thromboinflammation in hPAR4^Thr/Thr^ mice (Figure 6, F–J).

### Stroke outcomes in hPAR4^Thr/Thr^ mice are not improved by adding COX inhibition to P2Y12 blockade.

**Figure 7:**
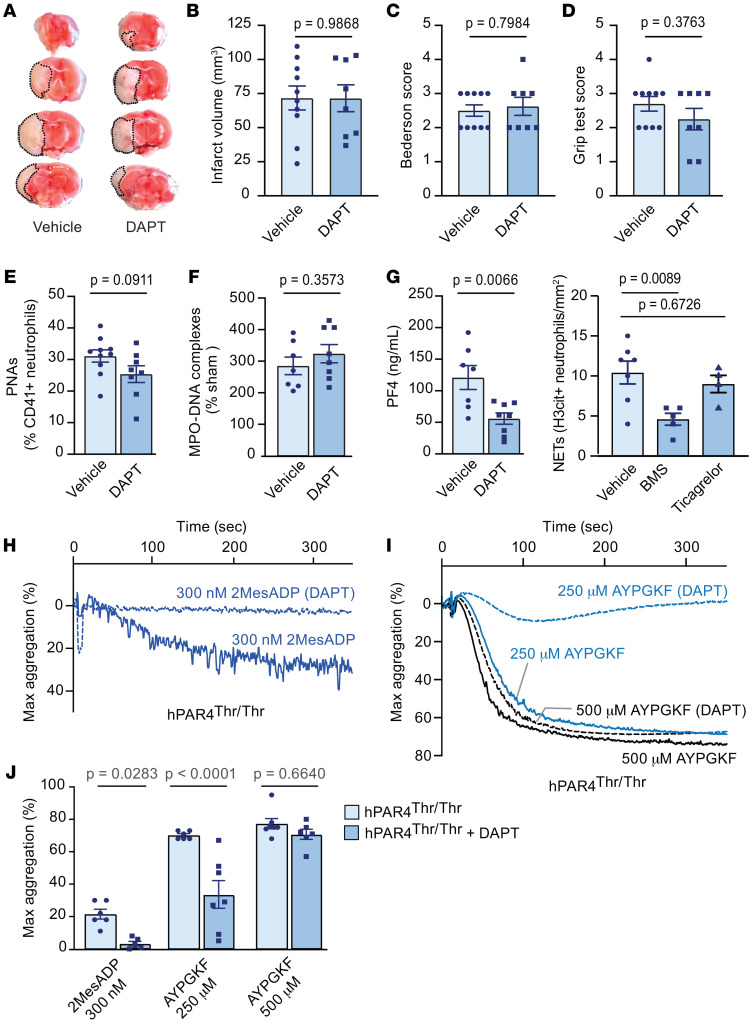
DAPT does not improve IS outcomes in hPAR4^Thr/Thr^ mice. Aspirin and ticagrelor or vehicle was administered by oral gavage to hPAR4^Thr/Thr^ mice 2 hours before tMCAO. Mice were then subjected to 60 minutes of tMCAO followed by 23 hours of reperfusion (severe). Whole blood, plasma, and brains were isolated 24 hours after stroke for analyses. (**A**) Brain infarct volumes were quantified by staining with TTC, which colors live brain tissue red, while dead brain tissue remains white (outlined with black dotted lines). Representative images from *n* = 7–8 per group. (**B**) Quantification of brain infarct volumes. Neurological and motor function were assessed by the Bederson (**C**) and grip (**D**) tests, respectively. Normality was determined by Shapiro-Wilk test, while significance was determined by an unpaired *t* test (**B**) and Mann-Whitney *U* test (**C** and **D**). *n* = 8–10. (**E**) PNAs were measured in mouse whole blood by flow cytometry by gating on Ly6G^+^ neutrophils that were positive for CD41^+^ platelets. *n* = 7–8 per group. (**F**) NET formation in mouse plasma was measured using an MPO-DNA ELISA. *n* = 7–8 per group. (**G**) Platelet activation as assessed by PF4 release was measured in mouse plasma by ELISA. *n* = 7–8. Normality was determined by Shapiro-Wilk test, while significance was determined by an unpaired *t* test (**E**–**G**). (**H**–**J**) DAPT or vehicle was administered by oral gavage 2 hours before platelets were isolated from hPAR4^Thr/Thr^ mice. Washed platelets were resuspended in Tyrode’s buffer, and the aggregation response to 2MesADP and AYPGKF was measured. (**H**) 2MesADP representative aggregation tracings from *n* = 6–7 per group. (**I**) AYPGKF representative aggregation tracings from *n* = 6–7 per group. (**J**) Quantification of all curves. Significance was determined by a mixed-effect analysis with a Šidák’s multiple-comparisons test. *n* = 6–7 per group.

Because hPAR4^Thr/Thr^ mice exhibited persistent platelet activation despite in vivo administration of a P2Y12 antagonist (Figure 6, F–H, and Supplemental Figure 21), we investigated whether adding aspirin to the regimen (DAPT, a standard approach in patients) would improve stroke outcomes. DAPT-treated hPAR4^Thr/Thr^ mice had similar IS brain injuries (Figure 7, A and B) and no improvement in either neurological behavior or motor function (Figure 7, C and D) compared with vehicle-treated animals. Circulating PNAs and NET levels of DAPT-treated hPAR4^Thr/Thr^ mice were not significantly different than those in vehicle-treated animals (Figure 7, E and F) despite having reduced platelet activation, as demonstrated by decreased circulating plasma PF4 (Figure 7G). DAPT was therapeutically effective based on decreased arachidonic acid–induced platelet aggregation (Supplemental Figure 22, A and B). While DAPT had little effect on stroke or stroke outcomes, DAPT treatment significantly increased tail-clip bleeding times in hPAR4^Thr/Thr^ mice (Supplemental Figure 22, C and D). Ex vivo PAR4-mediated aggregation using platelets from hPAR4^Thr/Thr^ mice revealed DAPT reduced platelet aggregation by 50% in response to low concentration AYPGKF, which was not observed after ticagrelor treatment alone (Figure 7, H–J). DAPT had no effect on aggregation at a high concentration of AYPGKF (Figure 7, I and J).

### P selectin blockade improves stroke outcomes in hPAR4^Thr/Thr^ mice.

**Figure 8:**
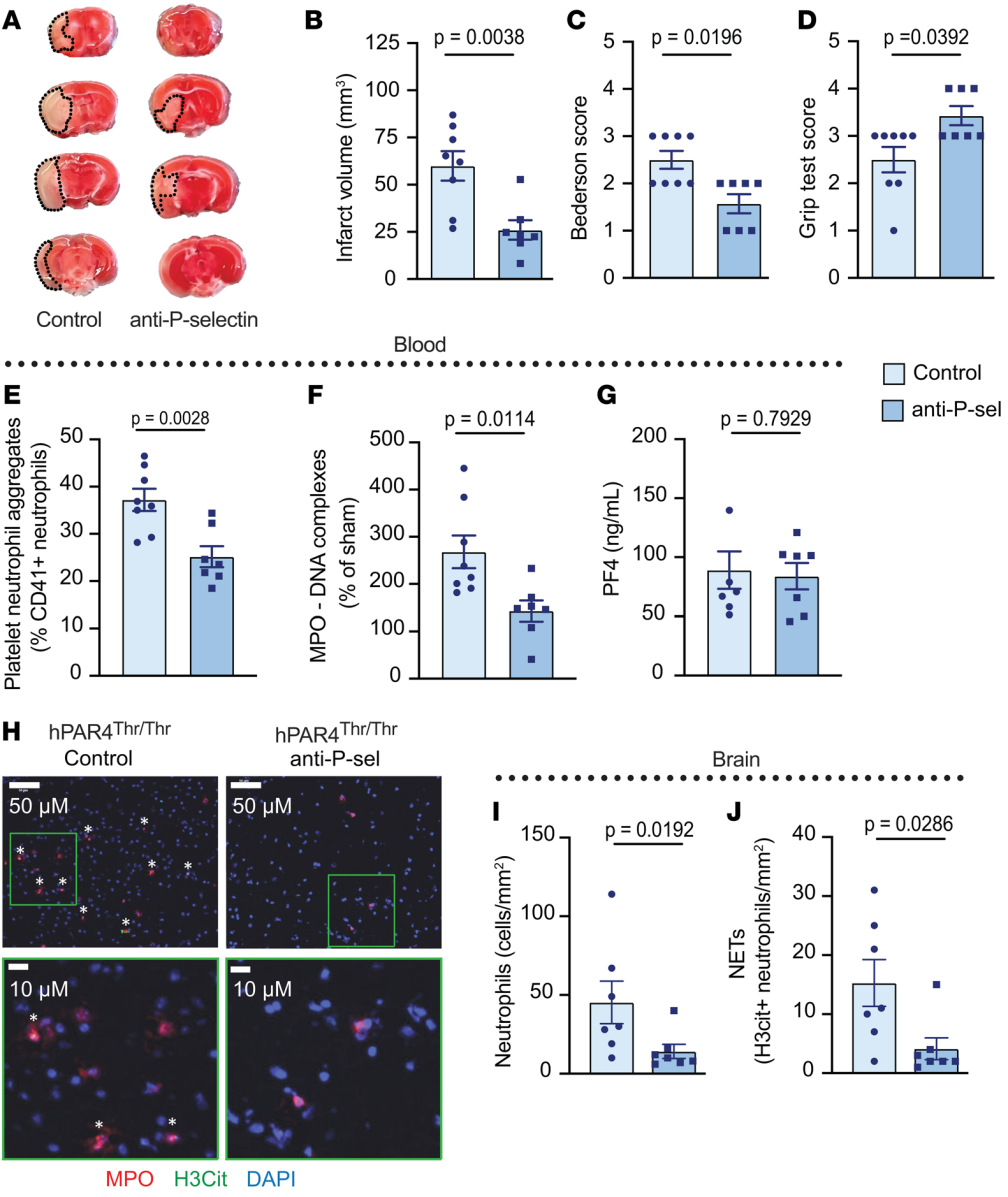
P selectin blockade improves IS outcomes in hPAR4^Thr/Thr^ mice. Anti–P selectin or control IgG (2 mg/kg) was administered i.v. to hPAR4^Thr/Thr^ mice 1 hour before tMCAO. Mice were subjected to 60 minutes of tMCAO followed by 23 hours of reperfusion (severe). (**A**) Brain tissue was stained with TTC. Dead brain tissue is outlined with black dotted lines. Representative images from *n* = 7–8 per group. (**B**) Quantification of brain infarct volumes. Neurological and motor function were assessed by the Bederson (**C**) and grip (**D**) tests. Normality was determined by Shapiro-Wilk test, while significance was determined by unpaired *t* test (**B**) and Mann-Whitney *U* test (**C** and **D**). *n* = 7–8. (**E**) PNAs were measured by flow. (**F**) NET formation in the plasma was measured using an MPO-DNA ELISA. (**G**) PF4 release was measured in plasma. (**H**) Representative images of stained neutrophils in infarcted brain tissue identified by MPO (red) and DNA (DAPI; blue). (**I**) Summary of the number of neutrophils per infarct area. Representative images from *n* = 6–8 per group. (**J**) Percentages of NET-forming neutrophils were quantified by counting H3cit-positive neutrophils. NETs are identified by the asterisks. Normality was determined by Shapiro-Wilk test, while significance was determined by an unpaired *t* test (**E**–**J**). *n* = 6–8 per group**.**

Together, these data suggest persistent platelet-neutrophil interactions despite standard antiplatelet treatment drives stroke outcomes in hPAR4^Thr/Thr^ mice. As platelet P selectin is a critical regulator of platelet-neutrophil interactions and anti–P selectin therapy was recently FDA approved for the treatment of sickle-cell disease, we assessed tested P selectin blockade in hPAR4^Thr/Thr^ mice subjected to stroke. Anti–P selectin treatment of hPAR4^Thr/Thr^ mice prior to tMCAO resulted in a greater than 50% reduction in brain infarct size compared with isotype control–treated animals (Figure 8, A and B). This reduced brain injury was accompanied by improved neurological and motor function (Figure 8, C and D). Anti–P selectin treatment also reduced circulating PNAs (Figure 8E) and plasma NETs (Figure 8F) without affecting platelet activation (Figure 8G). In addition, P selectin blockade resulted in a reduction in recruitment of brain neutrophils (Figure 8, H and I) and NETs (Figure 8, H and J) compared with what occurred in isotype-treated hPAR4^Thr/Thr^ mice. Similar protection by P selectin blockade was observed in hPAR4^Ala/Ala^ mice after tMCAO (Supplemental Figure 23). Notably, anti–P selectin therapy had no effect on tail-clip bleeding in hPAR4^Thr/Thr^ mice (Supplemental Figure 24).

### Impact of antiplatelet drugs on hemorrhagic transformation in hPAR4 Ala120 and hPAR4 Thr120 mice subjected to IS.

Platelets are critical drivers of IS brain injury and are essential for maintaining vascular integrity in the ischemic brain. Different platelet receptors have distinct effects on cerebral thrombosis and vascular integrity (32). To assess vascular integrity, coronal brain sections were examined for macroscopic hemorrhaging (Supplemental Figure 25). Results showed vehicle-treated hPAR4^Ala/Ala^ or hPAR4^Thr/Thr^ mice had low incidence of brain hemorrhaging (10.3% and 6.5%, respectively; Table 3). None of the treatment regimens increased brain hemorrhaging in hPAR4^Ala/Ala^ mice. However, ticagrelor with or without aspirin increased brain hemorrhaging in hPAR4^Thr/Thr^ mice, particularly in the high-dose ticagrelor and DAPT groups (Table 3). Both BMS-986120 and anti–P selectin treatment did not affect the incidence of brain hemorrhaging in hPAR4^Thr/Thr^ mice.

## Discussion

There is a limited understanding of biologic contributors to racial health disparities. This report sought to characterize the role of a racially divergent, hyperreactive protease-activated GPCR variant in an in vivo model of IS. Our major findings are as follows: (a) compared with hPAR4^Ala/Ala^ mice, hPAR4^Thr/Thr^ mice had worse outcomes; (b) the worse stroke outcomes in hPAR4^Thr/Thr^ mice were caused, at least in part, by enhanced platelet reactivity, enhanced PNA formation, and enhanced brain NET formation; (c) standard antiplatelet therapies (ticagrelor with or without aspirin) improved stroke outcomes in hPAR4^Ala/Ala^ mice, but not in hPAR4^Thr/Thr^ mice; (d) P selectin blockade reduced stroke size in hPAR4^Thr/Thr^ and hPAR4^Ala/Ala^ mice; and (e) the rs773902 AA genotype was a risk factor for IS and worse neurological outcomes in Black individuals. These findings demonstrate the functionality of the PAR4 Ala120Thr variant in vivo and ex vivo, underscore the pathophysiologic importance of PAR4-mediated platelet activation to induce NETs in stroke, and suggest a different therapeutic approach for stroke management in patients homozygous for the common hyperactive PAR4 Thr120 allele.

Understanding PAR4 function has long been a challenge due to poor-quality anti-PAR4 antibodies and confounding variables (e.g., demographic). Until recently, prior attempts to stably express human PARs in mouse platelets were unsuccessful, but Renna et al. used a transgenic approach to express hPAR4 and showed that, compared with WT mouse platelets, platelets from transgenic mice showed increased reactivity to the synthetic peptide AYPGKF, but less reactivity to human thrombin (33). This study did not consider the Ala120Thr variant effects, and these results underscore the need to assess hPAR4 effects on vascular disease in vivo. To directly test the in vivo functional effects of the PAR4 Ala120Thr variant unencumbered by demographic and other genetic variants and to develop an in vivo system for mechanistic and pharmacologic insights into hPAR4, we generated hPAR4^Ala/Ala^ and hPAR4^Thr/Thr^ mouse lines that had *F2rl3* replaced by a single copy of *F2RL3*. We observed similar expression of *F2RL3* and total hPAR4 protein in platelets and other tissues known to express PAR4 and demonstrated hPAR4 has a robust response to mouse thrombin, a requirement for a useful model of thrombin-PAR4–mediated in vivo thrombosis. While hPAR4 surface expression remains unknown in PAR4^Ala/Ala^ and PAR4^Thr/Thr^ mice due to challenges obtaining anti-hPAR4 antibodies for flow cytometry, our data suggest hPAR4 expression does not drive the differences in platelet hyperreactivity, consistent with human studies (16).

We specifically assessed the variant effect on platelets by ex vivo–washed platelet experiments. Our findings provide powerful support for the Thr120 allele affecting both platelet activation and aggregation. hPAR4 dependence on the strain difference in platelet function was demonstrated by both blockade with BMS-986120, an anti-PAR4 antagonist in phase 1 clinical trials (34), and the lack of genotype effect on collagen-induced platelet activation. Platelet aggregation data are consistent with findings of Whitley et al. showing a persistent genotype effect on platelet aggregation with only low but not high concentrations of thrombin (17).

Platelets play a critical role in both thrombus formation during the initial ischemia phase and neutrophil migration during the reperfusion phase of stroke (35). Since both human and our mouse data showed the Ala120Thr variant only altered ex vivo platelet aggregation at the steep part of the thrombin dose-response curve, we performed the tMCAO model under conditions that induced either moderate or severe ischemia. After moderate ischemia, hPAR4^Thr/Thr^ mice had larger infarcts and worse neurologic outcomes compared with hPAR4^Ala/Ala^ mice, demonstrating PAR4 Thr120 mediates an in vivo prothrombotic effect. Brain ischemia will induce the expression of thrombin, which is a key driver of secondary microthrombosis after blood flow is initially restored (36). The magnitude of thrombin generated depends on the duration and extent of ischemia (37). We speculate that there is a critical time after which the generated thrombin is at a low concentration at which platelets from hPAR4^Ala/Ala^ mice will not activate, but platelets from hPAR4^Thr/Thr^ mice will activate irreversibly, thus increasing the likelihood of secondary microthrombosis and subsequent reperfusion injury in Thr120 mice.

In addition to platelet-platelet interactions, (38) interactions between platelets and neutrophils are critical in mediating reperfusion injury contributing to IS (9, 39). Previous studies have demonstrated platelets expressing P selectin and PS (i.e., procoagulant platelets) serve as a nidus to mediate the formation of platelet-neutrophil macroaggregates (30, 31) and recruitment of neutrophils to the reperfused brain (29, 39). Platelets in these aggregates release soluble mediators to induce neurotoxic and prothrombotic NETs (10). Our data showed increased procoagulant platelet responses in PAR4^Thr/Thr^ mice compared with PAR4^Ala/Ala^ mice, consistent with prior in vitro studies (18), which likely facilitates the increased PNA formation observed in vivo (29, 39). We also demonstrate that platelets isolated from humans or mice carrying the Thr120 allele were more potent at inducing in vitro NETs. Due to a limited sample size of homozygous Thr humans, whether the number of copies of the Thr120 allele is additive for inducing NETs remains to be determined. We speculate that the combination of these factors contributes to the more severe in vivo stroke phenotype in which more brain NETs are observed in hPAR4^Thr/Thr^ mice.

After severe ischemia, both homozygous mouse strains had substantially larger infarcts and the difference in infarct size was lost, analogous to the findings in platelet aggregation studies with higher concentrations of thrombin. The large infarcts with the severe ischemic injury allowed assessment of PAR4 blockade, and infarct size was similarly reduced (40%–50%) in both homozygous strains. These experiments demonstrate stroke dependence on hPAR4 in this murine model, as has been reported using mPar4^–/–^ mice (40).

There is very limited data on the effect of aspirin and P2Y12 antagonists on ex vivo PAR4-induced platelet function. A single study testing for a pharmacogenetic effect with the PAR4 Ala120Thr variant found that Thr120-positive subjects continued to have higher ex vivo AYPGKF-induced platelet aggregation than Ala120-positive subjects taking either aspirin or clopidogrel for 7 days (19). These subjects were healthy volunteers, and demographic variables were not considered. In addition, studies using in vitro–added P2Y12 antagonists have yielded apparently conflicting results regarding the dependence of PAR4 on ADP-P2Y12 signaling in human platelets, but the Ala120Thr variant was not considered (41). The current report is what we believe to be the first pharmacogenetic study with an in vivo thrombotic outcome and shows that P2Y12 antagonism with oral ticagrelor improves stroke outcomes in hPAR4^Ala/Ala^ mice; however, under these same conditions, no benefit was observed in the hPAR4^Thr/Thr^ mice. The ex vivo aggregation data from these mice indicated PAR4-mediated platelet aggregation depends on P2Y12 signaling at low and high concentrations of PAR4 agonists, but only in hPAR4^Ala/Ala^ platelets. hPAR4^Thr/Thr^ platelet aggregation did not depend on P2Y12 signaling. Potential explanations for the genotype effect on aggregation include greater ADP release from hPAR4^Thr/Thr^ platelets, resistant PAR4 desensitization in hPAR4^Thr/Thr^ platelets, and/or greater signaling via PAR4 Thr120-P2Y12 heterodimers (17). A potential explanation for the genotype effect on stroke includes greater PNA and NET formation in the brains of hPAR4^Thr/Thr^ mice. Aspirin is commonly used with P2Y12 antagonists to prevent recurrent ischemic events. While DAPT reduced global platelet activation and aggregation in response to low-dose AYPGKF, adding aspirin to ticagrelor did not affect stroke outcomes in hPAR4^Thr/Thr^ mice, indicating PAR4^Thr/Thr^-dependent platelet activation during stroke was not dependent on P2Y12 or COX-2 pathways. However, aspirin combined with ticagrelor altered hemostasis and increased bleeding in the brain of hPAR4^Thr/Thr^ mice (Table 3), consistent with human clinical trials (42). Our results suggest oral, commonly used antiplatelet therapy with aspirin and P2Y12 antagonism improves stroke outcomes in hPAR4^Ala/Ala^ mice, but not hPAR4^Thr/Thr^ mice, and causes more brain bleeding in the latter. These findings raise hypotheses that require further human studies.

Since our findings support the importance of platelet-neutrophil interactions for worsening stroke outcomes in hPAR4^Thr/Thr^ mice, we tested the effect of blocking P selectin, a key regulator of platelet-neutrophil interactions. Pretreatment with a P selectin–blocking antibody substantially improved stroke outcomes in both hPAR4^Ala/Ala^ and hPAR4^Thr/Thr^ mice. The reduction in blood and brain NETs after tMCAO by P selectin blockade in treated hPAR4^Thr/Thr^ mice further supports the role for platelet-neutrophil interactions as a central regulator of IS. In addition, as endothelial cell P selectin is critical in IS (43), anti–P selectin therapy may target this in addition to platelet-neutrophil interactions. Finally, our data with hPAR4^Thr/Thr^ mice are similar to those in studies in sickle-cell disease mice, in which P selectin blockade reduced circulating PNAs and neutrophil activation and decreased lung vascular permeability (44). Unlike aspirin and ticagrelor, anti–P selectin did not alter hemostasis or bleeding in the brain of hPAR4^Thr/Thr^ mice (Table 3) (45).

When grouped by SNP markers, Black individuals, based on race/ethnicity, have a substantially higher frequency of the rs773902 A risk allele. In addition, based on race/ ethnicity, Black subjects have a higher risk of IS than White subjects (12–14) and evidence suggests Africa has up to 2- to 3-fold greater rates of stroke incidence than Western Europe and the US (46). To date, few studies have focused on candidate gene associations with disease in Black populations (47), and we are not aware of other candidate genetic association studies reporting on rs773902 as a stroke risk factor in Black subjects. Selvadurai et al. observed no effect of rs773902 on thrombotic complications in a trial of low-dose aspirin in predominantly White subjects (48). However, there was limited power to test a variant effect, since there were only 4 stroke patients with the AA genotype. Whitley et al. performed a retrospective analysis of publicly available NCBI databases, using only self-identified White subjects, and reported an association between the rs773902 A allele and IS (17). The current REGARDS study was prospective, included a large sample size (~7,600 participants) and long follow-up for stroke, with 487 cases, and evaluated functional outcome after stroke. Since the effect of one or two copies of the rs773902 A allele on a multifaceted clinical phenotype such as stroke is complex, multiple models were used to assess risk. We observed an increased risk of the rs773902 AA genotype with incident IS and immediate postneurologic outcome in a recessive model that is consistent with our murine studies. A similar trend was observed with an additive genetic model (Supplemental Tables 5 and 6). Notably, antiplatelet therapy data were not obtained at the time of IS in REGARDS, so pharmacogenetic analyses were not possible. Taken together, this longitudinal study of self-identified Black individuals, coupled with our murine studies, provides consistent support for the rs773902 AA genotype as a risk factor for IS. The AA genotype had a moderately low HR, quite typical of complex diseases, such as stroke (49, 50), and our data support a role for both PAR4 in the pathophysiology of IS and use of rs773902 in a polygenetic risk score for IS (50).

To begin to consider the effect of a single copy of the Thr120 allele, PAR4^Ala/Ala^ and hPAR4^Thr/Thr^ mice were crossed, generating hPAR4^Ala/Thr^ mice. In the moderate stroke model, hPAR4^Ala/Thr^ mice had an IS brain injury like that in hPAR4^Thr/Thr^ mice and greater than that in hPAR4^Ala/Ala^ mice (Supplemental Figure 26, A and B). However, when hPAR4^Ala/Thr^ mice were treated with ticagrelor, they were protected like hPAR4^Ala/Ala^ mice (Supplemental Figure 26, C–I). These data suggest a complex gene-dosage effect depending on multiple variables, and comprehensive studies are needed to address gene and antiplatelet dosage on IS outcomes.

There are several limitations to our study. First, the lack of PAR1 on murine platelets prevents drawing conclusions on the role of PAR4-PAR1 interactions in regulating additional effects of the variant on platelet hyperreactivity and IS risk and outcomes. The ability to experimentally examine this in vivo is hindered by the lack of murine models where PAR1 is expressed on murine platelets. While several groups have attempted to generate mice expressing human PAR1 on murine platelets, they have not been successful (51, 52). However, PAR4 but not PAR1 is responsible for (a) platelet PS exposure and microparticle release (53, 54), (b) fibrin formation under shear stress (18), and (c) NET formation (Figure 2K, Figure 3, and Supplemental Figure 15). Furthermore, the PAR4 Ala120Thr variant alters thrombin-induced human platelet aggregation, granule release, and platelet accumulation under arterial shear ex vivo when *both* PAR4 and PAR1 are present (17, 19). Thus, despite the limitation regarding PAR1-PAR4 interactions in this and many other murine thrombosis models, our data directly address the specific role of the PAR4 variant on platelet function and stroke. Second, although the tMCAO mouse model for studying stroke is commonly used, it does not recapitulate all aspects of human IS. In this model, IS is induced in all mice, thus making it difficult to infer the effect of the variant on stroke incidence in a human population. Third, we appreciate that the PAR4 Ala120Thr variant on other cell types, such as endothelial cells or neurons, may play a role in stroke pathophysiology. Finally, mouse platelets express both PAR3 and PAR4. Mouse PAR3 does not signal to activate platelets, but serves as a cofactor to present thrombin to PAR4. While our data do not address the efficiency of mouse PAR3/human PAR4 interactions, the ability for murine thrombin to cleave human PAR4 demonstrates mouse PAR3 is not required for human PAR4-mediated platelet activation, aggregation, and brain injury.

The findings here support consideration of possible new treatments and precision medicine approaches to patient care. Current antiplatelet agents reduce the risk of recurrent stroke, myocardial infarction, or death by only approximately 22% (55). In this regard, there are potential therapeutic implications of our work. First, PAR4 blockade may benefit people with either rs773902 allele. If effective, such interventions could improve racial disparities in stroke outcomes. Second, P selectin antagonism may be a valuable treatment for stroke prevention, particularly in high-risk PAR4 Thr120-positive patients, such as in sickle-cell disease, where approximately 40% and approximately 47% of patients are expected to have AA and AG genotypes, respectively. Notably, the P selectin antagonist crizanlizumab is FDA approved for prevention of sickle-cell anemia pain crises. Third, many more clinical studies are needed that include diverse patient populations. In the US, White subjects make up approximately 90% of the participants in clinical trials for cardiovascular therapeutics (56). Relevant to our report, the THALES trial with 11,000 participants demonstrated ticagrelor and aspirin were clinically superior to aspirin alone for the primary end points of stroke risk and death, but only enrolled 52 Black subjects (42). Our work emphasizes that drugs and dosing that are based primarily on one segment of the population (e.g., White patients primarily expressing the PAR4 Ala120 variant) may not be the optimal drug or dosing for another group (e.g., any patients primarily expressing the PAR4 Thr120 variant).

## Methods

### REGARDS study

The REGARDS study is a population-based, longitudinal cohort study of 30,239 Black and White American adults 45 years or older from all 48 contiguous US states and the District of Columbia (57). By design, participants were oversampled if they were residents of the stroke belt or if they were Black. REGARDS is investigating the reasons why the stroke mortality is higher among Black compared with White adults as well as residents of the Southeast US compared with other regions. At enrollment in 2003–2007, participants completed a computer-assisted telephone interview to collect demographic information and an in-home visit for blood pressure measurements and collection of blood and urine specimens. IRBs at each participating REGARDS institution approved the REGARDS protocol (57).

We included all Black participants with available genotyping for rs773902 through a REGARDS ancillary GWAS study (58). We excluded participants with anomalies in informed consent, who were not part of the ancillary genetic study, who were White, who had a self-reported history of stroke, who were missing data on rs773902, and who were lacking stroke follow-up. An inclusion flow diagram for the study is provided in Supplemental Figure 16.

#### Incident stroke definition.

Participants were contacted at 6-month intervals to obtain information on possible incident stroke. The medical records associated with these events were retrieved and adjudicated by a committee using the WHO definition of stroke as having focal neurologic symptoms lasting more than 24 hours or neuroimaging data consistent with stroke (59, 60). ISs were further classified into etiologic subtypes of SVO, large artery atherosclerosis (LAA), CE, other, or undetermined (61). mRS immediately after stroke was available from medical records in a subset of cases (~51%). The mRS is a 7-level, clinician-reported measure of global disability, ranging from 0 (no symptoms) to 6 (death) (62, 63). All incident ISs occurring before or on September 30, 2020, were included in this analysis. REGARDS participants with a history of a previous stroke were excluded.

#### Genotyping, imputation, and quality control of rs773902.

Genotyping was performed on Black participants using the Illumina (Illumina Inc.) Expanded Multi-Ethnic Global Array (MEGA^EX^) as part of an ancillary study in REGARDS (58). For the current study, only variant rs773902 was retained and had an imputation quality score (Rsq) of 98% and an effect allele (A) frequency of 0.546. The final quality-controlled sample after exclusions (described above and in Supplemental Figure 16) included 7,620 Black participants (487 incident IS cases and 7,133 noncases).

### Generation of humanized mice

A hPAR4 C57BL/6 mouse was generated by excising *F2rl3* and inserting an *F2RL3* sequence with rs773902 G (hPAR4^Ala^) in its place using the *Easi-*CRISPR method (22, 64). The *Easi-*CRISPR method uses CRISPR ribonucleoproteins (to cleave the genome at the desired site or sites) and a long single-stranded DNA (lssDNA) containing short homology arms (to serve as a template for knocking in the sequence at the genomic cut sites). Two guide RNAs, one each cleaving at the start and stop codon of *F2rl3*, were used. The sequence for the *F2rl3* locus was derived from the ensembl.org database (https://uswest.ensembl.org/Mus_musculus/Transcript/Exons?db=core;g=ENSMUSG00000050147;r=8:73488508-73490502;t=ENSMUST00000058099). The upstream guide (CTGATCCTGGCAGCATG/TGC) cleaves immediately after the start of the codon, and the downstream guide (CTCCTCTACACTTCTGT/GAC) cleaves within the stop codon. The start and stop codons are marked as bold-faced letters, and the guide cleavage sites are shown with a slash. A custom lssDNA was commercially obtained from IDT (65). C57BL6/J mouse zygotes were used for CRISPR microinjection, and the mice were generated following the protocols described previously (64, 66). Genotyping screens of the founder mice were performed using junctional PCRs in which one of the primers binds to the genomic sequence outside the homology arm and the other primer binds within the insert. Genotyping of G0 generation mice showed 3 potential positive founders of which founder mouse no. 3 was used for breeding to establish the hPAR4^Ala^ line. The hPAR4^Thr^ knockin mouse line was established using offspring of the founder hPAR4^Ala^ mouse no. 3 and the CRISPR methodology as described above, except replacing F2RL3 with rs773902. Heterozygous hPAR4^Ala/Thr^ mice were generated by crossing hPAR4^Ala/Ala^ mice with hPAR4^Thr/Thr^ mice.

### Ex vivo NET formation in humans and mice

For human NET assays, healthy human donors aged between 18 years and 50 years from the greater Salt Lake City (Utah, USA) area with race self-identified were screened and genotyped for rs773902 as previously described (17). Donors were age and sex matched for the NET assays.

### Murine IS model

tMCAO was performed as described previously (10, 26, 29, 30). Briefly, occlusion of the right MCA was achieved by inserting a standardized monofilament (Doccol Corp.) via the right internal carotid artery to occlude the origin of the right MCA. The occluding suture was left in situ for variable lengths of time.

### Statistics

#### REGARDS cohort.

Baseline descriptive statistics for incident IS cases and noncases were presented as counts (percentages) for categorical variables or mean ± SD for continuous variables (Supplemental Tables 1–4). The risk allele, A, for rs773902 was modeled for (a) additive, (b) dominant (AA+AG versus GG), and (c) recessive (AA versus AG+GG) genetic inheritance in all analyses. We calculated HRs with 95% CIs of IS using Cox’s proportional hazards models to describe the association between rs773902 and incident IS. A base dose-dependent model was adjusted for age, sex, and the first 5 genetic PCs. Additional models were adjusted for cigarette smoking, hypertension (defined as systolic blood pressure [BP] ≥140 mmHg or diastolic BP ≥ 90 mmHg or self-reported current medication use to control BP), and type 2 diabetes (defined as fasting glucose ≥126 mg/dL or nonfasting glucose ≥200 mg/dL or taking pills or insulin for self-reported diabetes).

Case-only analyses among IS cases were performed for 269 participants with data for mRS to examine the relationship between rs773902 and functional outcomes. mRSs of 0 to 2 were defined as a favorable functional outcome and considered the referent, while scores of 3 to 6 were indicative of unfavorable functional outcomes. All statistical analyses were performed using SAS, version 9.4 (SAS Institute Inc.).

According to REGARDS policy, the hypotheses, aims, and analysis plan for this manuscript were prespecified and reviewed and approved by the REGARDS publications committee, which also reviewed the final manuscript and assured the a priori plans were followed.

#### Laboratory experiments.

These statistical analyses were performed with GraphPad Prism, version 9.1.2 (GraphPad Software). Prior to analysis, a Shapiro-Wilks test was used to check data distributions. Two-sided unpaired *t* tests or ANOVA (1 or 2 way where indicated) with indicated post hoc tests were used for statistical comparison when applicable. In the case of nonparametric data, a Mann-Whitney *U* test or Kruskal-Wallis test with post hoc Dunn’s correction was performed. All data are represented as dot plots, including a bar graph with error bars representing mean ± SEM. A *P* value of less than 0.05 was considered statistically significant.

### Study approval

The primary research goals were to examine the in vivo effect of the PAR4 variant rs773902 on IS and determine whether various antiplatelet therapies were effective based on genotype. IS burden in humanized mice under various treatments was assessed using the tMCAO stroke. All animal experiments complied with the regulatory standards of the University of Utah (IACUC 21-09012) and were performed following the ARRIVE guidelines, including randomization and analysis blind to the genotype. All experiments were performed using 8- to 12-week-old male and female mice. The following conditions excluded mice from end-point analyses (exclusion criteria): (a) death within 12 hours after tMCAO; (b) operation time greater than 10 minutes; and (c) surgical complications. IRBs at each participating REGARDS institution approved the REGARDS protocol. All donors provided informed consent based on approval from the University of Utah IRB (IRB 00095539).

### Data availability

WGS data can be found at the NIH’s Sequence Read Archive database (SRA PRJNA995031). Values for all data points in graphs are reported in the Supporting Data Values file.

## Author contributions

FD, NDA, MLS, IP, EAT, RMDT, EM, SB, M Cody, JLR, AA, NDT, DCM, JB, MTN, SEM, CCY, LAL, M Cushman, MRI, PFB, and RAC designed and performed experiments. FD, NDA, M Cushman, MRI, and RAC analyzed results and made the figures. FD, MC, PFB, and RAC wrote the manuscript. All authors reviewed and critically edited the manuscript.

## Supplementary Material

Supplemental data

Supporting data values

## Figures and Tables

**Table 2:**
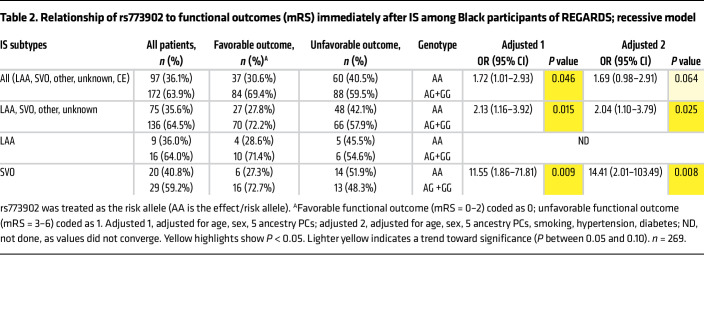
Relationship of rs773902 to functional outcomes (mRS) immediately after IS among Black participants of REGARDS; recessive model

**Table 1:**
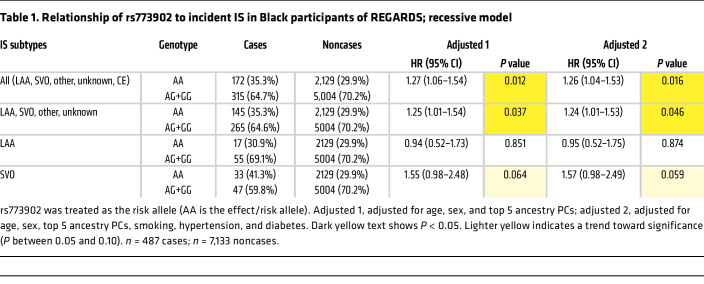
Relationship of rs773902 to incident IS in Black participants of REGARDS; recessive model

**Table 3:**
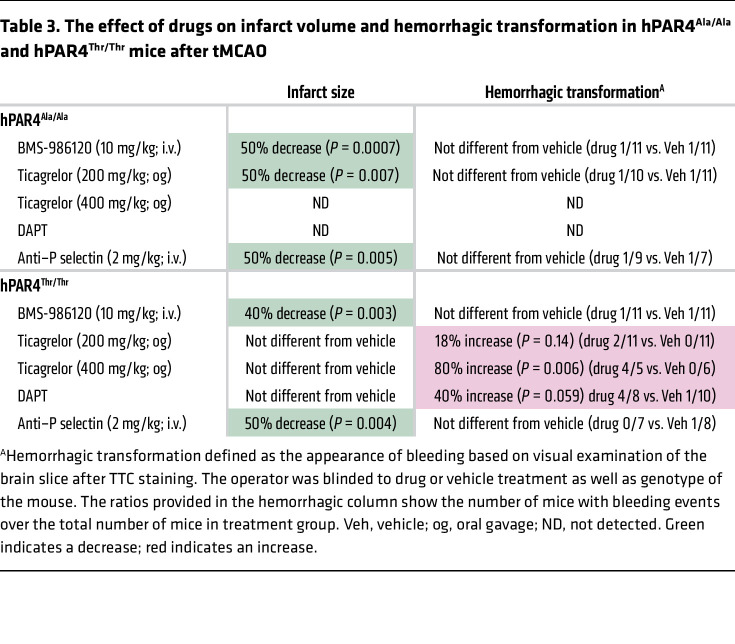
The effect of drugs on infarct volume and hemorrhagic transformation in hPAR4^Ala/Ala^ and hPAR4^Thr/Thr^ mice after tMCAO

## Version 1 07/20/2023

In-Press Preview

## Version 2 09/15/2023

Electronic publication
