# Supplementary material for: The predominant PAR4 variant in individuals of African ancestry worsens murine and human stroke outcomes
Source: J Clin Invest. 2023 Sep 15;133(18):e169608. doi: 10.1172/JCI169608 (PMC10503801; doi:10.1172/JCI169608)
Supplement: Supplemental data [file jci-133-169608-s185.pdf]

The predominant PAR4 variant in individuals of African ancestry worsens murine and human stroke outcomes

Frederik Denorme[1,2], Nicole D. Armstrong[3], Michelle L. Stoller[1], Irina Portier[1], Emilia A. Tugolukova[1], Rikki M. D. Tanner[3], Emilie Montenont[1], Seema Bhatlekar[1], Mark Cody[1], John L. Rustad[1], Abigail Ajanel[1], Neal D. Tolley[1], Darian C. Murray[1], Julie Boyle[4], Marvin T. Nieman[5], Steven E. McKenzie[6], Christian Con Yost[1,7], Leslie A. Lange[8], Mary Cushman[9], Marguerite R. Irvin[2], Paul F. Bray[1,10], Robert A. Campbell[1,10,11]

[1]Program in Molecular Medicine, University of Utah, Salt Lake City, Utah, USA
[2]Department of Neurology, Division of Vascular Neurology, University of Utah, Salt Lake City, USA
[3]Department of Epidemiology, University of Alabama at Birmingham, Birmingham, Alabama, USA.
[4]Bioinformatics Shared Resource, Huntsman Cancer Institute, University of Utah, Salt Lake City, UT, USA, 84132
[5]Department of Pharmacology, Case Western Reserve University, Cleveland, Ohio, USA.
[6]Department of Medicine, The Cardeza Foundation for Hematologic Research, Thomas Jefferson University, Philadelphia, Pennsylvania, USA.
[7]Department of Pediatrics, Division of Neonatology, University of Utah, Salt Lake City, UT, 84112, USA.
[8]Division of Biomedical Informatics and Personalized Medicine, Department of Medicine, University of Colorado Anschutz Medical Campus, Aurora, Colorado, USA.
[9]Department of Medicine, Larner College of Medicine at the University of Vermont, Burlington, VT, USA
[10]Department of Internal Medicine, Division of Hematology and Hematologic Malignancies, University of Utah, Salt Lake City, Utah, USA
[11]Department of Pathology, Division of Microbiology and Immunology, University of Utah, Salt Lake City, Utah, USA

The authors have declared that no conflict of interest exists

Running Title: Human PAR4 variant Thr120 and Stroke

Corresponding Author:
Robert Campbell, PhD
Assistant Professor of Medicine
University of Utah Health Sciences Center
Eccles Institute of Human Genetics
15 North 2030 East, Bldg 533, Suite 4280
Salt Lake City, Utah 84112
801-585-0702
rcampbell@u2m2.utah.edu

SUPPLEMENTAL MATERIALS

REGARDS Genotyping, Imputation, and Quality Control (QC) of rs773902- Genotyping was performed on Black participants using the Illumina (Illumina Inc, San Diego, CA) Expanded Multi-Ethnic Global Array (MEGA^{EX}) as part of an ancillary study in REGARDS.(1) Imputation was conducted using release 2 of the National Heart Lung and Blood Institute (NHLBI) Trans-omics for Precision Medicine (TOPMed) reference panel available through BioData Catalyst.(2) Participants were excluded if they had sex mismatches (self-report versus genetic), had a genotyping call rate <0.95, were duplicates, or an outlier (± 6 standard deviations) in the principal component (PC) analysis.(3) For the current study, only variant rs773902 was retained and had an imputation quality score (Rsq) of 98% and an effect allele (A) frequency of .546. The final QC'd sample after exclusions (described above and **Supplemental Fig. 12**) included 7,620 Black participants (487 incident IS cases and 7,133 noncases).

Whole genome sequencing in mice

DNA from the tail for genotyping and sequencing was isolated and extracted from both hPAR4^{Ala} and hPAR4^{Thr} using TRIS-HC and EDTA. DNA was treated with RNase and then measured using a Qubit dsDNA HS Assay. DNA libraries were prepped by the University of Utah Bioinformatics Core using a Nextera DNA Flex Library Prep with Unique dual indexes (UDI) and whole genome sequencing (WGS) was performed using the NovaSeq with 150 base-pair paired reads and 200 million reads per sample. Adapters were trimmed from fastqs using cutadapt(4) and alignments were completed using Burrows-Wheeler Aligner (BWA)(5) against the human hg38 reference fasta and the mouse mm10 reference fasta. The resulting bam files were sorted and indexed via samtools(6) and de-duplicated with picard. Picard was also used to generate insert size and WGS metrics for quality analysis. Additional quality reports were generated using MultiQC.(7) Alignments of *F2RL3* were viewed for accuracy and completeness in Integrative Genomics Viewer (IGV).(8) To pull all read pairs containing at least 25 base pairs of *F2RL3* for supplementary alignment and analysis, Seqkit(9) was used to filter reads from sample fastqs using intra-gene primer binding site sequences and 25bp kmers. The resulting reads were aligned against the *F2RL3* inserted sequence to verify completeness of the insertions. Additionally, the locations of the 5' and 3' sequence anchors were determined via BLAT(10) of hybrid hg38/mm10 sequence read pairs against the mm10 genome in IGV.(8)

PAR4 Expression Analysis in mice

mRNA. To assess platelet *F2RL3* expression, CD45 bead-depleted platelets were isolated from hPAR4$^{Ala/Ala}$ and hPAR4$^{Thr/Thr}$ mice as previously described and platelets were lysed in

Trizol.(11) RNA was extracted and cDNA generated as described.(11) Brain, liver, and lungs were also isolated from $hPAR4^{Ala/Ala}$ and $hPAR4^{Thr/Thr}$ mice and lysed in Trizol. RNA was extracted and cDNA generated as described.(11) Primers against *F2RL3* (forward – GCTGCTGCATTACTCGGAC and reverse – ACGTAGGCACCATAGAGGTTG) were used with *Gapdh* as a housekeeping gene (forward – AGGTCGGTGTGAACGGATTTG and reverse – TGTAGACCATGTAGTTGAGGTCA).

Restriction Enzyme Digestion. DNA was isolated as described above from $hPAR4^{Ala/Ala}$ and $hPAR4^{Thr/Thr}$ mice and amplified using primers spanning *F2RL3* (forward – CCTTGGCCCAGTTCTTTATGAG and reverse CAGACGTATAGTACCCAGACC). After amplification, *NruI* (New England Biolabs, Ipswich, MA) was added for 30 minutes at 37°C. Digestion products were visualized on an agarose gel.

Protein. Platelet lysates from $hPAR4^{Ala/Ala}$ and $hPAR4^{Thr/Thr}$ mice were immunoblotted using a polyclonal antibody raised against a peptide corresponding to the cytoplasmic domain of hPAR4.(12)

PAR4 Knockout mice

PAR4 global knockout mice were a gift from Dr. Satya Kunapuli (Temple University, Philadelphia, PA) and Dr. Steven McKenzie (Thomas Jefferson University, Philadelphia, PA).(13)

Tail-Clip Bleeding Assay

Tail clip bleeding assays were performed as described before with limited modifications.(14) Briefly, anesthesia was induced by inhalation of 5% isoflurane and maintained by inhalation of 2% isoflurane. Four millimeters of the tail end was transected and immediately placed in warm PBS. The time until bleeding stopped was measured for initial and complete (defined as ceased bleeding for >1 min) bleeding cessation. If bleeding did not cease after ten minutes, the experiment was terminated. To quantify blood loss, the number of red blood cells collected during the bleeding was counted using a Hemavet (Drew Scientific; Miami Lakes, FL).

Platelet aggregation, immunoblotting and platelet activation in mice

Platelet aggregation. Platelets were isolated from whole blood for all experiments, as described previously.(11, 15-17) Whole blood was drawn from 8–12-week-old mice by cardiac puncture into 10% Acid Citrate Dextrose. Platelet isolation was performed as previously described.(11, 15, 18) Whole blood was diluted to 2 mL with warmed (37°C) pipes saline glucose (PSG; 5 mM PIPES, 145 mM NaCl, 4 mM KCl, 50 µM Na_2HPO_4, 1 mM $MgCl_2$ and 5.5

mM Glucose, pH 6.8) and centrifuged at 150 *x g* for 10 minutes to separate the platelet rich-plasma (PRP). The PRP was removed and prostaglandin E1 (PGE1; Cayman Chemical, Ann Arbor, MI) was added to the PRP to prevent exogenous platelet activation. The PRP was then centrifuged at 400 *x g* for 10 minutes at room temperature. A second wash with warmed PSG in the presence of PGE1 was performed. Platelets were resuspended in Tyrode's buffer (0.137 M NaCl, 2.68 mM KCl, 0.485 mM NaH_2PO_4, 12mM $NaHCO_3$, 5 mM HEPES, 500 μM $CaCl_2$, 250 μM $MgCl_2$, 5.5 μM Glucose and 52.7 nM BSA; pH 7.35) at 2 x 10^8 platelets/mL and aggregation was measured at 37°C using a PAP-8E aggregometer after stimulation with AYPGKF (PAR4-specific agonist; GL Biochem Ltd., Shanghai, China), human α-thrombin (Sigma, Darmstadt, Germany) or collagen (Chrono-log, Havertown, PA). BMS-986120 (10 mg/kg; Cayman) and anti-P-selectin antibody (2 mg/kg; BD) were administered intravenously 1 hour before platelet isolation. Ticagrelor (200 mg/kg; Selleck Chemicals, Houston, TX) and aspirin (20 mg/kg; University of Utah pharmacy) were administered in water through oral gavage 2 hours before experiments. In some experiments, after aggregation in response to AYPGKF, platelet proteins were precipitated with perchloric acid and centrifuged to examine Akt signaling. Platelet lysates were washed with water twice before being resuspended in Lamelli buffer. Western blots were performed as previously described and blots were probed with p-AKT-S473 (9271, Cell Signaling Technology, Danvers, MA) and total AKT (2920S, Cell Signaling Technology) antibodies. In addition, Fibrinogen levels in platelets were probed with DAKO rabbit anti-fibrinogen antibody (A0080).

Platelet activation. After agonist stimulation with AYPGKF, human α-thrombin, murine thrombin (Innovative Research, Novi, MI) or convulxin (CVX; Santa Cruz, Dallas, TX), platelet activation was measured in washed platelets by staining platelets with APC-labelled rat anti-mouse CD41 (MWREG30; ThermoFisher, Waltham, MA), FITC-labelled anti-P-selectin (Wug.E9; Emfret, Würzburg, Germany) and PE-labelled JON/A antibody (M023-2, Emfret). Procoagulant platelet (Annexin V positive) formation was induced by co-stimulation with human α-thrombin and CVX and detected by APC-labelled rat anti-mouse CD41 and FITC-labelled annexin V (ThermoFisher). Samples were then analysed on a Beckman Coulter Cytoflex (Beckman-Coulter, Pasadena, CA) located in the Utah Flow Cytometry Core

Murine ischemic stroke model

All animal experiments complied with the regulatory standards of the University of Utah (IACUC 21-09012) and were performed following the ARRIVE guidelines (www.nc3rs.org.uk), including randomization and analysis blind to the genotype. All experiments were performed using 8-12-week-old male and female mice.

Transient middle cerebral artery occlusion stroke model. Transient middle cerebral artery occlusion (tMCAO) was performed as described previously.(15-18) Briefly, occlusion of the right MCA was achieved by inserting a standardized monofilament (Doccol Corp, Sharon, MA) via the right internal carotid artery to occlude the origin of the right MCA. The occluding suture was left *in situ* for variable lengths of time. Induction of IS was confirmed by neurological testing of the mice while the MCA was occluded. Anesthesia was induced by inhalation of 5% isoflurane and maintained by inhalation of 2% isoflurane. Buprenorphine (University of Utah pharmacy) was administered one hour before surgery and every 12 hours as needed. Sham surgery was performed similarly, without insertion of the monofilament. The following conditions excluded mice from endpoint analyses (exclusion criteria): (1) death within 12 hours after tMCAO, (2) operation time > 10 minutes or (3) when surgical complications occurred. Brains of dead mice were visually checked for surgical complications and stained with 2,3,5-triphenyl-tetrazolium chloride (TTC, T8877; Sigma) when possible, as described below, to confirm IS-related mortality. For murine studies, mice were excluded based on pre-specific exclusion criteria, which involved a surgical bleed during the transient middle cerebral artery model.

Neurological and motor scoring. Twenty-four hours post stroke onset, mice were subjected to the modified Bederson test and the grip test to assess neurological and motor function, respectively.(15-18)

Determination of brain infarct size. To quantify IS brain damage, 2-mm-thick coronal brain sections were stained with 2% TTC to distinguish unaffected brain tissue from infarcted tissue, 24 hours after stroke induction.(15-18) Stained slices were photographed and infarct areas (white) were measured using Image J software (National Institutes of Health; Bethesda, MD) by an operator blinded to genotype and treatment.

Histology of murine ischemic stroke brains

Twenty-four hours after the tMCAO, mice were euthanized, the brains were dissected, snap-frozen in OCT compound and stored at -80°C until cryo-sectioning into 10 μm slices. Before immunohistology staining, slides were fixed in 4% paraformaldehyde and blocked in 3% donkey serum with 0.5% Tween20. Primary antibodies were goat anti-MPO (2 μg/mL, AF3667; R&D Systems, Minneapolis, MN) and rabbit anti-human citrullinated Histone H3 (2 μg/mL, ab5103; Abcam, Cambridge, United Kingdom). Secondary antibodies were AF488-labeled donkey anti-rabbit (2 μg/mL, R37118; ThermoFisher) and AF546-labeled donkey anti-goat (2 μg/mL, A-11056; ThermoFisher). DAPI was used as a nuclear counterstain (300nM,

D1306; Life Technologies, Carlsbad, CA). Images were acquired using a high-resolution, confocal reflection microscope (Olympus IX81, FV300; Olympus, Tokyo, Japan).

Platelet-neutrophil aggregate formation

Blood was collected and diluted 1:10 into M199 supplemented with 100 U/mL heparin (University of Utah pharmacy). Diluted blood was left resting or activated with different concentrations of AYPGKF for 15 minutes. For the detection of platelet-neutrophil aggregates, diluted blood was stained with APC-labelled rat anti-mouse CD41 and BV510-labeled rat anti-mouse Ly6G (1A8, Biolegend, San Diego, CA).(16-18) Samples were fixed with FACS lysis buffer, centrifuged at 500 *x g* for 10 minutes and resuspended in PBS before analysis on a Beckman Coulter Cytoflex located in the Utah Flow Cytometry Core.

Transmission Electron Microscopy

For ultrastructural analyses, platelets were adhered to Acylar coated with poly-L-lysine and fixed in 2.5% glutaraldehyde in PBS and processed as previously described(19). The samples were subsequently washed and postfixed with 2% osmium tetroxide, rewashed, dehydrated by a graded series of acetone concentrations (50%, 70%, 90%, 100%; 2 × 10 minutes each), and embedded in Epon. Thin sections were counterstained (ie, uranyl acetate and lead citrate), viewed with a JEOL JEM-1011 electron microscope, and digital images were captured with a side-mounted Advantage HR CCD camera (Advanced Microscopy Techniques). Platelets were imaged and alpha granules counted. Alpha granules in at least 125 individual platelets were counted per independent experiment. The number of granules per independent experiment were averaged together to generate the number of granules per independent experiment.

ELISAs

Platelet Factor 4 (PF4) – Plasma samples were diluted 1:200 and PF4 was measured following the manufacturer instructions (ab202403, Abcam). Isolated platelets were lysed by freeze-thawing three times followed by shear through a 27-gauage needle. Platelet lysates were centrifuged at 12000xg to remove cellular debris and the analyzed.

MPO-DNA complexes – An *in-house* ELISA was used to quantify MPO-DNA complexes.(18, 20, 21) Briefly, after overnight coating with anti-MPO antibody (2 µg/ml; 0400-0002; Bio-Rad, Hercules, CA) at 4°C, a 96-well plate was blocked with 2.5% bovine serum albumin in PBS for 2 hours at room temperature. The plate was subsequently washed, before incubating for 90 minutes at room temperature with 20% human or mouse plasma in blocking buffer. The plate was washed five times, and then incubated for 90 minutes at room temperature with anti-DNA

antibody (1:10; Cell Death detection ELISA, 11544675001, Sigma). After five washes, the plate was developed with TMB substrate (T0440; Sigma).

***Ex vivo* NET formation in humans and mice**

For human NET assays, healthy human donors aged between 18 years and 50 years of self-identified race from the greater Salt Lake City, Utah area were screened and genotyped for rs773902 as previously described.(22) All donors provided informed consent based on approval from the University of Utah IRB (IRB 00095539). Donors were age and gender matched for the NET assays. Neutrophils were isolated from freshly collected whole blood of healthy adults or adult mice using the EasySep Direct Human Neutrophil Isolation kit (19666; Stemcell Technologies, Vancouver, Canada) or EasySep Mouse Neutrophil Enrichment Kit (19762; Stemcell Technologies), respectively, with greater than 95% purity.(18) Neutrophils were resuspended to a concentration of 1 x 10^6 cells/mL in Medium 199 (ThermoFisher). Platelets were purified as described previously(18, 20, 21), resuspended to 1 x 10^8 cells/mL in Medium 199 and activated for 15 minutes. Activated platelets and neutrophils were incubated at a 100:1 ratio to induce NETs for 2.5 hours at 37°C in 5% CO_2/95% air. NET levels were measured using an MPO-DNA ELISA as described above.

Supplemental Table 1. Blood cell counts in hPAR4$^{Ala/Ala}$ and hPAR4$^{Thr/Thr}$ mice.

Blood Parameter	hPAR4$^{Ala/Ala}$	hPAR4$^{Thr/Thr}$	P value
Platelets (K/μL)	968.7±32.2	948.2±15.0	0.571
Mean Platelet Volume	4.74±0.04	4.75±0.04	0.862
White Blood Cells (K/μL)	11.38±1.86	10.56±1.07	0.509
Neutrophils (K/μL)	1.91±0.27	2.01±0.19	0.748
Red Blood Cells (M/μL)	10.10±0.13	10.25±0.17	0.498

n=10 per group ± SEM

Supplemental Table 2. Baseline characteristics of REGARDS study participants.

Characteristic	Overall, N (%)	Incident Ischemic Strokes[1], N (%)	Non-cases, N (%)
N	7620	487 (6.4%)	7133 (93.6%)
Age, y			
45-54	1124 (14.8%)	37 (7.6%)	1087 (15.2%)
55-64	3174 (41.7%)	146 (30.0%)	3028 (42.5%)
65-74	2312 (30.3%)	206 (42.3%)	2106 (29.5%)
≥75	1010 (13.3%)	98 (20.1%)	912 (12.8%)
Sex			
Male	2930 (38.5%)	201 (41.3%)	2729 (38.3%)
Female	4690 (61.6%)	286 (58.7%)	4404 (61.7%)
HTN	5356 (70.4%)	399 (82.1%)	4957 (69.6%)
SBP, mmHg, mean ± SD	131 ± 17	135 ± 18	130 ± 17
DBP, mmHg, mean ± SD	78 ± 10	79 ± 10	78 ± 10
Smoker ever	4140 (54.6%)	274 (56.4%)	3866 (54.4%)
Diabetes	2135 (28.5%)	189 (39.4%)	1946 (27.8%)
P2Y12B Inhibitor	241 (3.16%)	20 (4.11%)	221 (3.10%)
Aspirin	2062 (27.06%)	167 (34.29%)	1895 (26.57%)

[1] Ischemic stroke cases occurred on or before September 30, 2020.

Supplemental Table 3. Baseline aspirin and P2Y12 inhibitor use by genotype in REGARDS.

	AA	GA	GG	P
P2Y12B Inhibitor	76 (31.54%)	117 (48.55%)	48 (19.92%)	0.899
Aspirin	605 (26.29%)	1047 (27.81%)	410 (26.38%)	0.347

[1] Ischemic stroke cases occurred on or before September 30, 2020.

Supplemental Table 4. Genotype of ischemic stroke cases by TOAST classification in REGARDS Black participants.

rs773902	LAA, n (%)	SVO, n (%)	CE, n (%)	Other, n(%)	Unknown, n (%)	Sum, n (%)
AA	17 (30.9%)	33 (41.2%)	27 (35.1%)	8 (27.6%)	87 (35.4%)	172 (35.3%)
GA	27 (49.1%)	33 (41.2%)	30 (39.0%)	20 (69.0%)	116 (47.0%)	227 (46.6%)
GG	11 (20.0%)	14 (17.5%)	20 (26.0%)	1 (3.4%)	43 (17.4%)	89 (18.2%)
Sum	55 (100%)	80 (100%)	77 (100%)	29 (100%)	247 (100%)	487 (100%)

LAA – Large-artery atherosclerosis

SVO – Small vessel occlusion

CE – Cardioembolic

Supplemental Table 5. Relationship of rs773902 with *incident ischemic stroke* in Black participants of REGARDS (n=487 cases, 7133 noncases) using an additive and dominant model.

Ischemic Stroke Subtypes	Model	Genotype	Cases	Noncases	Adjusted 1[2]		Adjusted 2[3]	
					HR (95% CI)	P value	HR (95% CI)	P value
All (LAA + SVO + Other + Unknown + CE)	Additive[1]	AA	172 (35.3%)	2129 (29.9%)	1.15 (1.01-1.31)	0.037	1.13 (1.00-1.32)	0.053
		AG	226 (46.4%)	3539 (49.6%)				
		GG	89 (18.3%)	1465 (20.5%)				
	Dominant	GG + AG	315 (64.7%)	5004 (70.2%)	1.09 (0.91-1.55)	0.457	1.07 (0.85-1.36)	0.553
		AA	172 (35.3%)	2129 (29.9%)				
LAA + SVO + Other + Unknown	Additive[1]	AA	145 (35.3%)	2129 (29.9%)	1.17 (1.01-1.35)	0.034	1.16 (1.01-1.35)	0.044
		AG	196 (47.8)	3539 (49.6%)				
		GG	69 (16.8%)	1465 (20.5%)				
	Dominant	GG + AG	265 (64.6%)	5004 (70.2%)	1.19 (0.91-1.55)	0.200	1.18 (0.90-1.55)	0.225
		AA	145 (35.3%)	2129 (29.9%)				
LAA	Additive[1]	AA	17 (30.9%)	2129 (29.9%)	0.95 (0.64-1.42)	0.818	0.96 (0.64-1.43)	0.833
		AG	27 (49.0%)	3539 (49.6%)				
		GG	11 (20.0%)	1465 (20.5%)				
	Dominant	GG + AG	55 (69.1%)	2129 (29.9%)	0.94 (0.47-1.88)	0.854	0.94 (0.47-1.88)	0.852
		AA	17 (30.9%)	5004 (70.2%)				
SVO	Additive[1]	AA	33 (41.3%)	2129 (29.9%)	1.26 (0.91-1.76)	0.170	1.26 (0.91-1.70)	0.167
		AG	33 (41.3%)	3539 (49.6%)				
		GG	14 (17.5%)	1465 (20.5%)				
	Dominant	GG + AG	47 (59.8%)	2129 (29.9%)	1.08 (0.60-1.94)	0.798	1.07 (0.60-1.92)	0.820
		AA	33 (41.3%)	5004 (70.2%)				

Note: rs773902 treated as the risk allele
[1]Hazard ratio for each additional copy of A allele
[2]Adjusted 1: adjusted for age + sex + top 5 ancestry PCs
[3]Adjusted 2: adjusted for age + sex + top 5 ancestry PCs + smoking + hypertension + diabetes
LAA - large-artery atherosclerosis
SVO – small vessel occlusion
CE – cardioembolic
Yellow highlights P < 0.05. Lighter yellow indicates a trend toward significance (P between 0.05 and 0.10)

Supplemental Table 6. Relationship of rs773902 with functional outcomes (modified Rankin scores, mRS) immediate post- ischemic stroke among Black participants of REGARDS **(N=269)** using an additive and dominant model.

Ischemic Stroke Subtypes	Genetic Inheritance Model	Genotype	All pts, n (%)	Favorable Outcome, n (%)[1]	Unfavorable Outcome, n (%)	Adjusted 1[2]		Adjusted 2[3]	
						OR (95% CI)	P value	OR (95% CI)	P value
All (LAA + SVO + Other + Unknown + CE)	Additive[1]	AA	97 (36.1%)	37 (30.6%)	60 (40.5%)	1.28 (0.89-1.8)	0.183	1.23 (0.86-1.76)	0.261
		AG	120 (44.6%)	61 (50.4%)	59 (39.9%)				
		GG	52 (19.3%)	23 (19.0%)	29 (19.6%)				
	Dominant	AA + AG	217 (80.7%)	98 (81.0%)	119 (80.4%)	0.99 (0.52-1.89)	0.977	0.93 (0.48-1.79)	0.822
		GG	52 (19.3%)	23 (19.0%)	29 (19.6%)				
LAA + SVO + Other + Unknown	Additive[1]	AA	75 (35.6%)	27 (27.8%)	48 (42.1%)	1.48 (0.99-2.21)	0.055	1.43 (0.95-2.16)	0.084
		AG	95 (45.0%)	50 (51.6%)	45 (39.5%)				
		GG	41 (19.4%)	20 (20.6%)	21 (18.4%)				
	Dominant	AA + AG	170 (80.6%)	77 (79.4%)	93 (81.6%)	1.21 (0.59-2.49)	0.596	1.16 (0.56-2.43)	0.689
		GG	41 (19.4%)	20 (20.6%)	21 (18.4%)				
LAA	Additive[1]	AA	9 (36.0%)	4 (286%)	5 (45.6%)	Did not converge due to sample size in cells			
		AG	11 (44.0%)	5 (35.7%)	6 (54.6%)				
		GG	5 (20.0%)	5 (35.7%)	0 (0.0%)				
	Dominant	AA + AG	20 (80.0%)	9 (64.3%)	11 (100.0%)				
		GG	5 (20.0%)	5 (35.7%)	0 (0.0%)				
SVO	Additive[1]	AA	20 (40.8%)	6 (27.3%)	14 (51.9%)	2.71 (0.96-7.64)	0.060	2.97 (0.98-8.98)	0.053
		AG	19 (38.8%)	12 (54.6%)	7 (25.9%)				
		GG	10 (20.4%)	4 (18.2%)	6 (22.2%)				
	Dominant	AA + AG	39 (79.6%)	18 (81.8%)	21 (77.8%)	1.22 (0.23-6.53)	0.818	1.29 (0.23-7.28)	0.772
		GG	10 (20.4%)	4 (18.2%)	6 (22.2%)				

Note: rs773902 treated as the risk allele
[1]Hazard ratio for each additional copy of A allele
[2]Adjusted 1: adjusted for age + sex + top 5 ancestry PCs
[3]Adjusted 2: adjusted for age + sex + top 5 ancestry PCs + smoking + hypertension + diabetes
LAA - large-artery atherosclerosis
SVO – small vessel occlusion
CE – cardioembolic
Yellow highlights P < 0.05. Lighter yellow indicates a trend toward significance (P between 0.05 and 0.10)

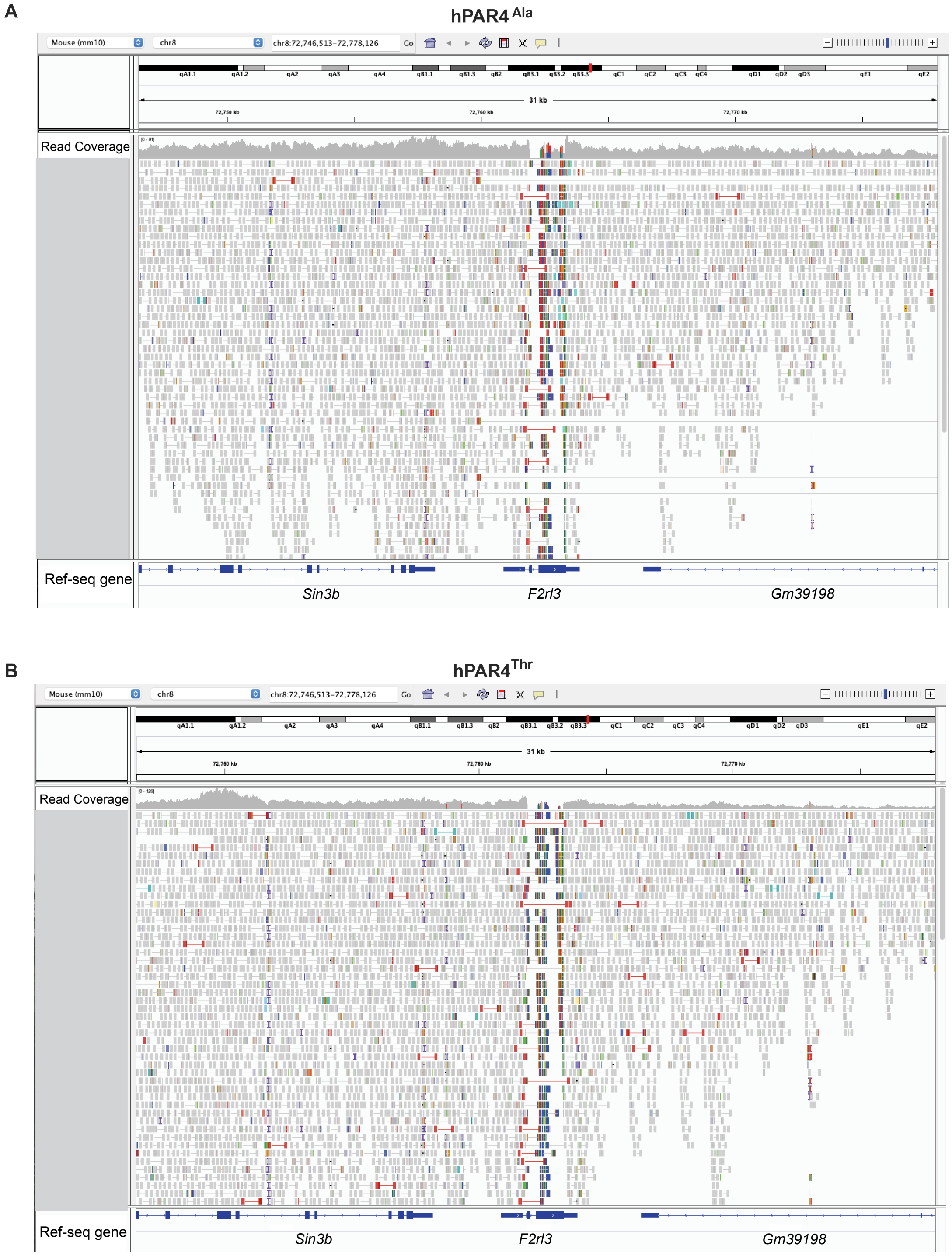

Supplemental Figure 1. Proper insertion of *F2RL3* into *F2rl3* locus. Whole Genome sequencing (WGS) was performed on the founder mouse from both the (**A**) hPAR4^{Ala} and (**B**) hPAR4^{Thr} lines. Human sequencing reads were aligned against the mm10 genome in IGV. Paired-end human reads are identified by colored reads. Reads that align against the mouse genome are in grey. (**A**) In the hPAR4^{Ala} founder mouse, the full-length *F2RL3* gene was present and was inserted at the targeted 5' and 3' junction sites. The *F2RL3* allele inserted in this locus was different by one SNP (at the expected variant nucleotide) compared to the

hPAR4^{Thr} mouse. WGS identified heterogeneity in the hPAR4^{Ala} founder mouse at the *F2rl3* locus as both human *F2RL3* and mouse *F2rl3* were present. (**B**). The full-length *F2RL3* coding region was present and was inserted at the targeted 5' and 3' junction sites in the hPAR4^{Thr} founder mouse. The hPAR4$^{Thr/Thr}$ founder mouse was homogeneous for human *F2RL3* as no mouse *F2rl3* was present. Both lines were backcrossed eight generations onto C57BL/6J. After eight generational backcrosses, heterozygous hPAR4 mice were crossed with each other to generate homozygous hPAR4$^{Ala/Ala}$ and hPAR4$^{Thr/Thr}$ mice.

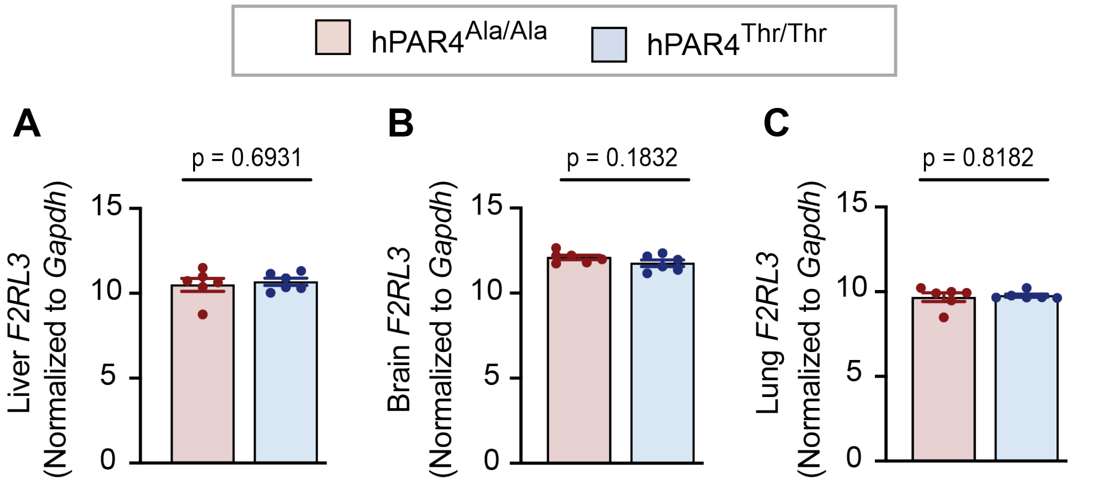

Supplemental Figure 2. *F2RL3* tissue expression is similar between hPAR4$^{Ala/Ala}$ and hPAR4$^{Thr/Thr}$ mice. *F2RL3* expression was assessed by qRT-PCR using mRNA isolated liver (**A**), brain (**B**) and lung (**C)** from hPAR4$^{Ala/Ala}$ and hPAR4$^{Thr/Thr}$ mice. N=6 per group. Normality determined by Shapiro-Wilk Test. Significance determined by unpaired T-tests (**A-B**) and Mann-Whitney test (**C**).

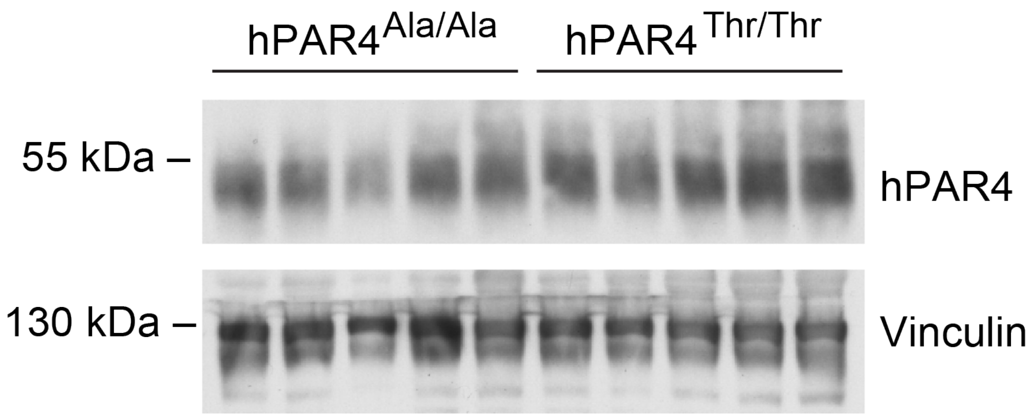

Supplemental Figure 3. Platelet PAR4 expression is similar between hPAR4$^{Ala/Ala}$ and hPAR4$^{Thr/Thr}$ mice. Platelets from hPAR4$^{Ala/Ala}$ and hPAR4$^{Thr/Thr}$ mice were stained for human PAR4 (upper blot). Platelet PAR4 protein levels in hPAR4$^{Ala/Ala}$ and hPAR4$^{Thr/Thr}$ mice were quantified and are shown in **Fig. 1F**. N=5 per group.

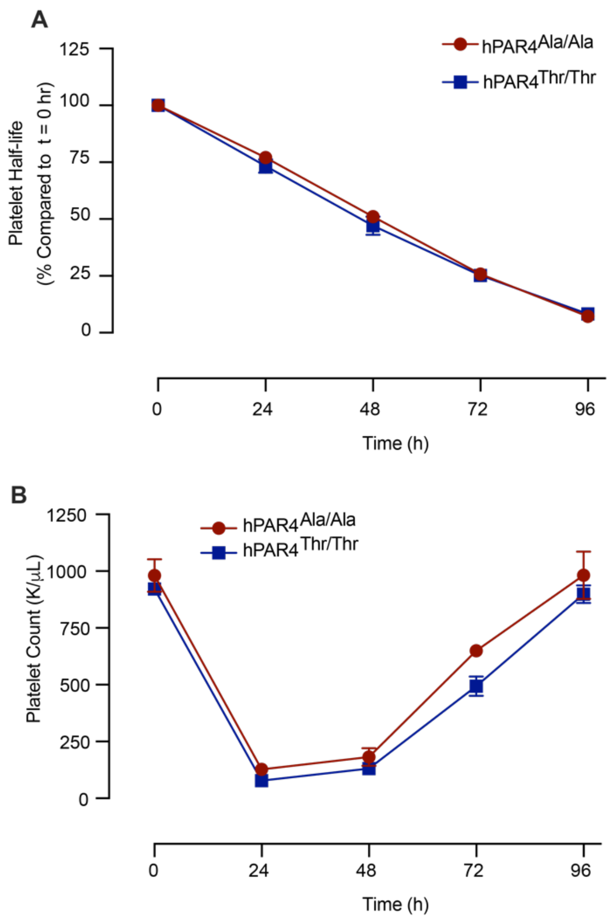

Supplemental Figure 4. Platelet half-life and production are comparable between between hPAR4$^{Ala/Ala}$ and hPAR4$^{Thr/Thr}$ mice. (**A**) hPAR4$^{Ala/Ala}$ and hPAR4$^{Thr/Thr}$ mice were inject intravenously with Dylight 488 anti-GPIbβ antibody. Four hours later whole blood was drawn by tail poke and platelets were labeled with anti-CD41 antibody. The labeled platelet population was set to a 100% based on the percentage of CD41-positive, Dylight-positive platelets as analyzed by flow cytometry. This was set as time = 0. The percentage of the remaining CD41-positive, Dylight-positive platelets was measured subsequently every 24 hours and divided by the percentage of CD41-positive, Dylight-positive platelets at time = 0. We observed no statistical difference at any time point between hPAR4$^{Ala/Ala}$ and hPAR4$^{Thr/Thr}$ mice. N=4 mice per group. Significance determined by 2-way ANOVA with a Sidak's multiple comparisons test. (**B**) Whole blood platelets counts were measured in hPAR4$^{Ala/Ala}$ and hPAR4$^{Thr/Thr}$ mice before mice were injected intravenously with an anti-GPIbα (2 μg/g) in PBS to deplete platelets. Subsequently, whole blood platelet counts were measured every 24 hours to measure platelet production. We observed no significant difference between hPAR4$^{Ala/Ala}$ and hPAR4$^{Thr/Thr}$ mice at any time point measured. N=4-5 mice per group. Significance determined by 2-way ANOVA with a Sidak's multiple comparisons test.

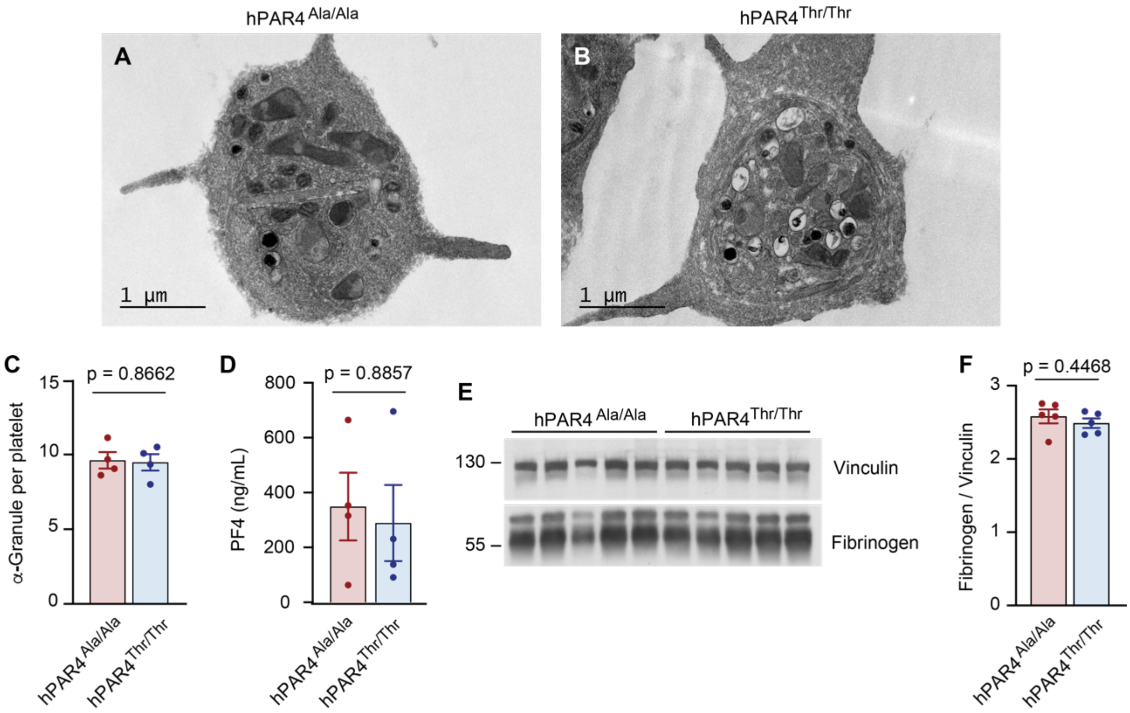

Supplemental Figure 5. Platelet morphology and granule contents are similar between hPAR4$^{Ala/Ala}$ and hPAR4$^{Thr/Thr}$ mice. (A-C) Washed platelets were isolated from hPAR4$^{Ala/Ala}$ and hPAR4$^{Thr/Thr}$ mice and placed on poly-l-lysine coated aclar. Platelets were fixed with paraformaldehyde and glutaraldehyde and prepared for transmission electron microscopy. Platelet alpha granule and morphology was examined. Alpha granules from more than 30 platelets per sample were counted. N=4 samples per group. Normality determined by Shapiro-Wilk Test. Significance determined by unpaired T-tests. (**D**) Washed platelets were isolated from hPAR4$^{Ala/Ala}$ and hPAR4$^{Thr/Thr}$ mice, and lysed using three freeze-thaw cycles and shear through a 21-gauge needle. Platelet factor 4 (PF4) was measured by ELISA. N=4 per group. Normality determined by Shapiro-Wilk Test with significance determined by Mann-Whitney test (**E-F**). Washed platelets were isolated from hPAR4$^{Ala/Ala}$ and hPAR4$^{Thr/Thr}$ mice and lysed. Fibrinogen levels were examined and quantified using densitometry of western blots. N=5 per group. Normality determined by Shapiro-Wilk Test. Significance determined by unpaired T-tests**.**

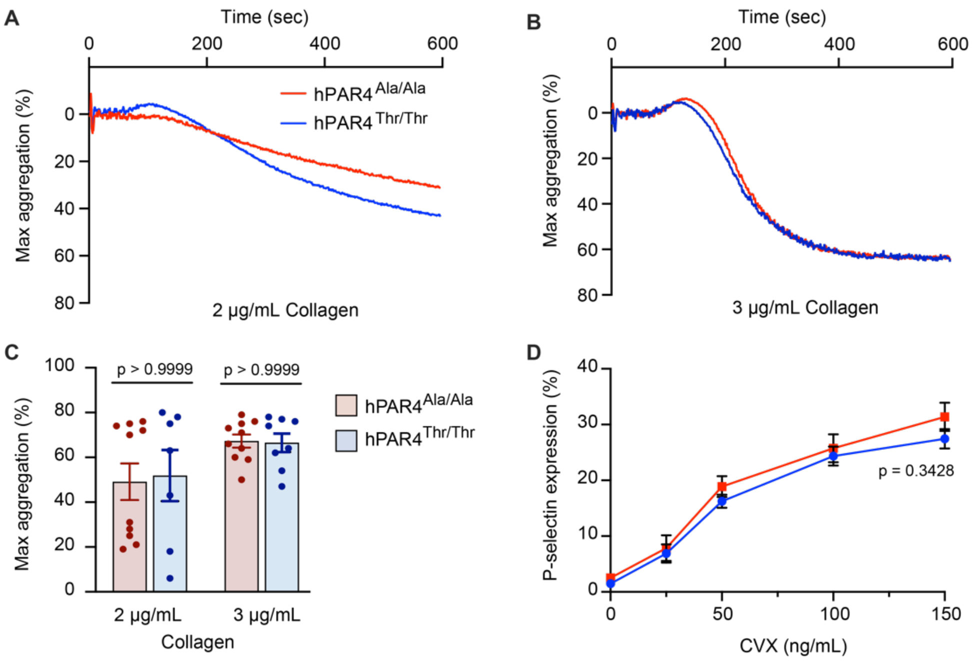

Supplemental Figure 6. Platelet aggregation and activation in response to GPVI agonists is similar between hPAR4$^{Ala/Ala}$ and hPAR4$^{Thr/Thr}$ mice. Washed platelets were isolated from hPAR4$^{Ala/Ala}$ and hPAR4$^{Thr/Thr}$ mice, resuspended in Tyrode's buffer at 2x10^8 platelets/mL, and stimulated with collagen at the indicated concentrations. Representative tracings for collagen at 2 μg/mL (**A**) and 3 μg/mL (**B**) are shown from N=8-11 per group. (**C**) Quantification of collagen-induced platelet aggregation data. N=7-10 per group. Significance determined by Mixed-effect ANOVA with a Bonferroni's multiple comparisons test. (**D**) Quantification of convulxin (CVX)-induced platelet activation data. N=8-11 per group. Significance determined by Mixed-effect ANOVA.

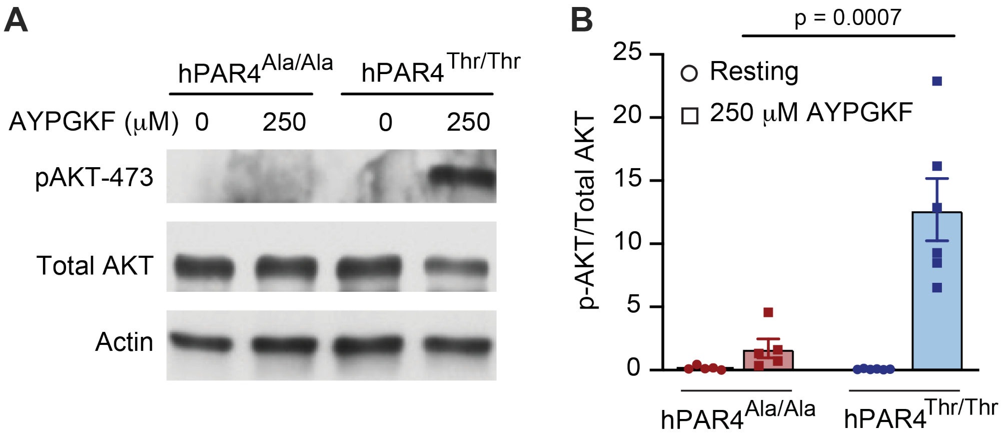

Supplemental Figure 7. hPAR4 activation induces greater Akt signaling in hPAR4$^{Thr/Thr}$ mice. Washed platelets were isolated from hPAR4$^{Ala/Ala}$ and PAR4$^{Thr/Thr}$ mice and stimulated with vehicle (resting, 0 μM PAR4-AP) or 250 μM PAR4-AP for five minutes and solubilized. (**A**) Platelet lysates were separated by SDS-PAGE and immunoblotted with and antibody against pAKT-473, total AKT and actin. A representative blot from an N=6 per group is shown. (**B**) Densitometric quantification of p-AKT normalized to total AKT. N=6 per group. Significance determined by Mixed effect ANOVA with a Bonferroni's multiple comparisons test.

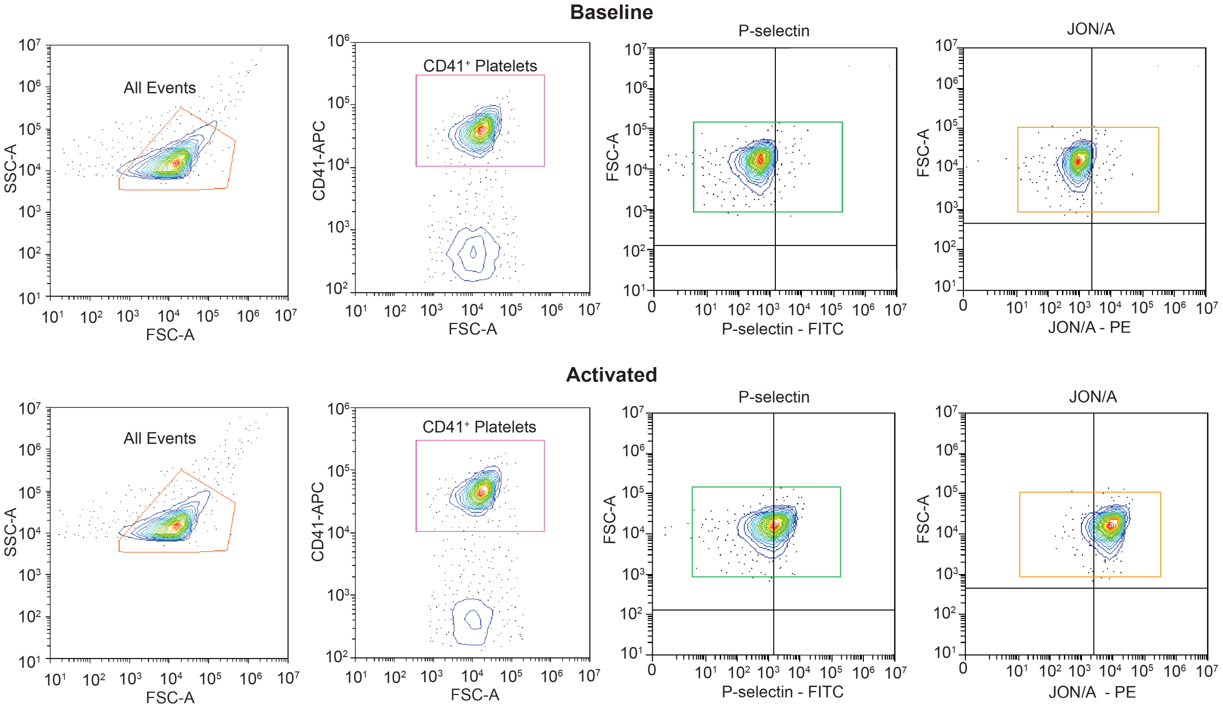

Supplemental Figure 8. Gating strategy to measure JON/A binding and P-selectin expression after platelet activation. Representative flow plots depicting JON/A binding and P-selectin expression from washed platelets at baseline or after activation from hPAR4$^{Ala/Ala}$. JON/A binding and P-selectin expression were defined as events in the upper right quadrant. A minimum of 10,000 total CD41 positive events were counted to determine expression.

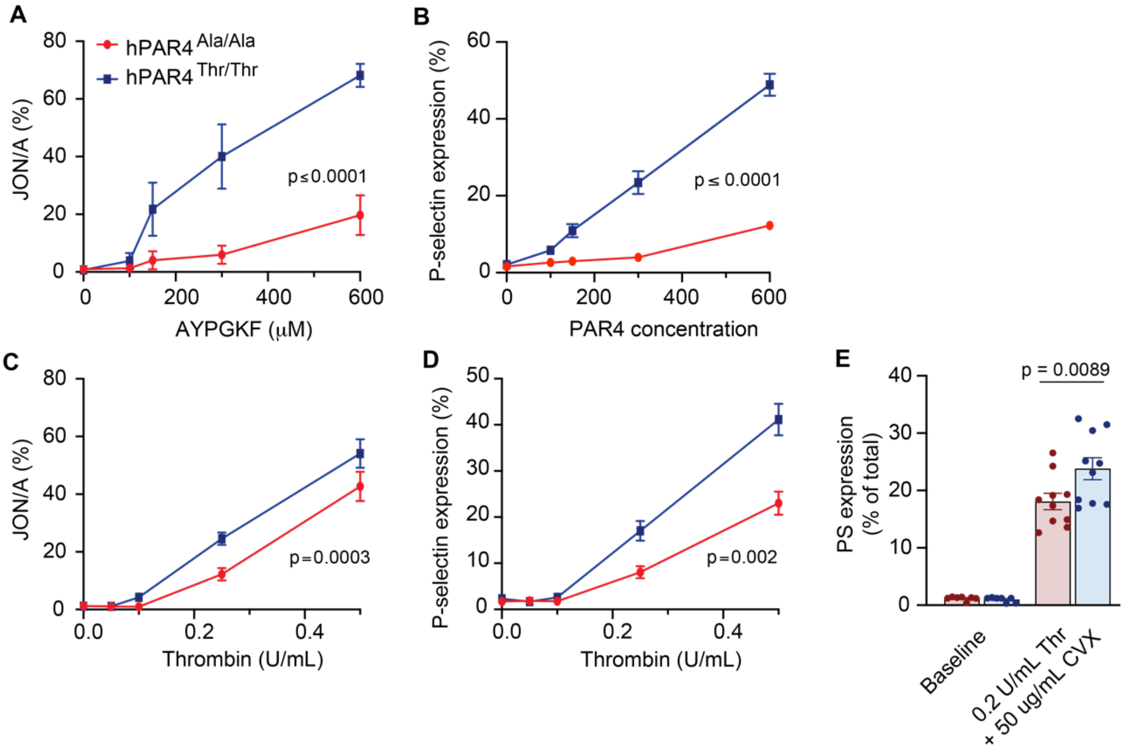

Supplemental Figure 9. Platelets from humanized PAR4$^{Thr/Thr}$ mice display greater agonist-induced activation than platelets from humanized PAR4$^{Ala/Ala}$ mice. Washed platelets isolated from hPAR4$^{Ala/Ala}$ and hPAR4$^{Thr/Thr}$ mice were stimulated with AYPGKF (**A, B**), thrombin (**C, D**) or thrombin plus convulxin (CVX) (**E**) at the indicated concentrations. Platelet activation was assessed by flow cytometric assessment of (**A, C**) integrin activation (JON/A binding), (**B, D**) alpha granule release (anti-P-selectin antibody binding) and (**E**) phosphatidylserine exposure (Annexin V binding on CD41+ platelets). N=3-10 per group for AYPGKF; N=3-10 per group for thrombin; N = 10 per group for thrombin + CVX. Significance determined by Mixed effect ANOVA with a Sidak's multiple comparisons test (**A-E**).

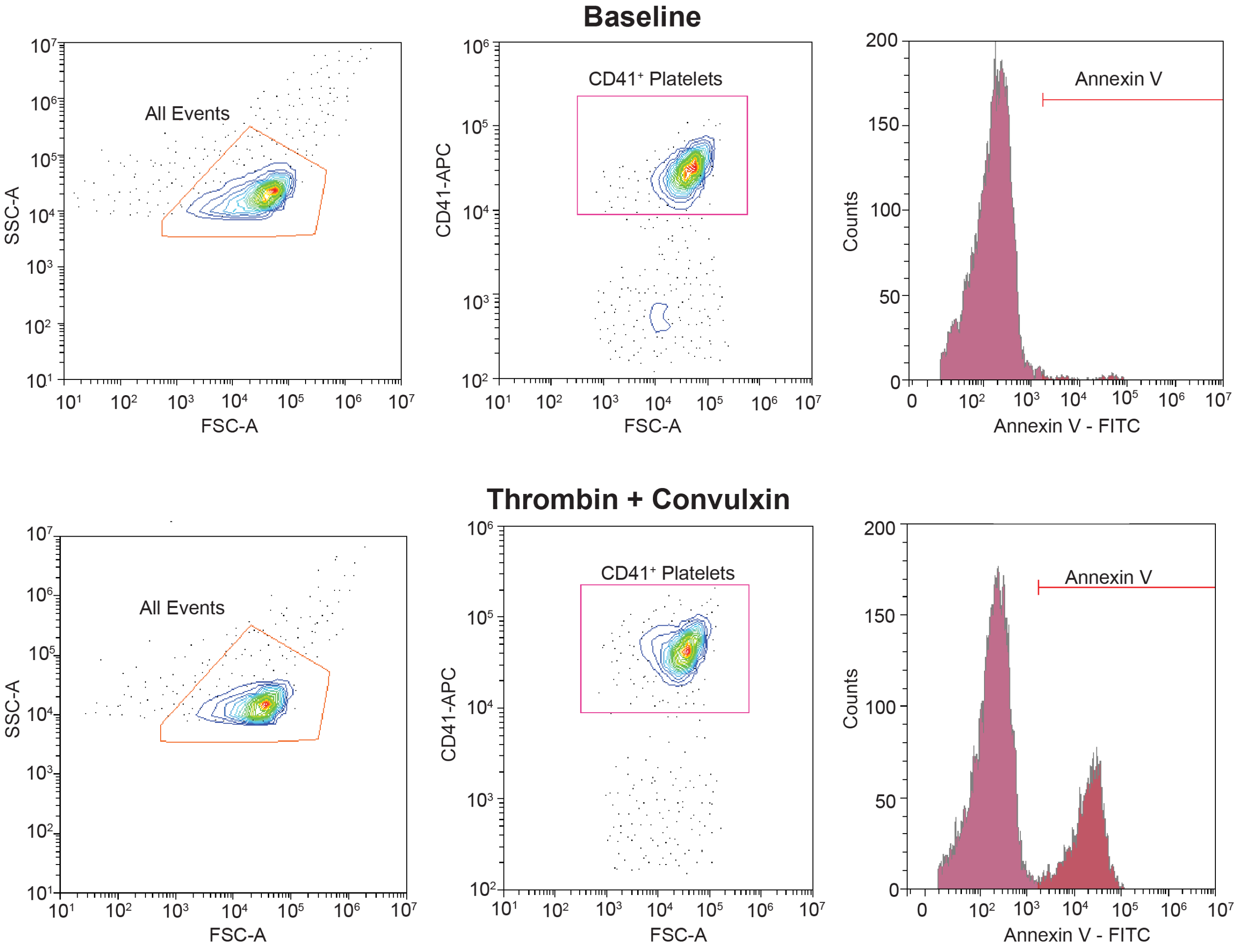

Supplemental Figure 10. Gating strategy to measure procoagulant platelet formation. Representative flow plots depicting PS exposure based on Annexin V binding on washed platelets at baseline or after thrombin and convulxin stimulation in hPAR4$^{Thr/Thr}$. A minimum of 10,000 total CD41 positive events were counted to determine PS expression.

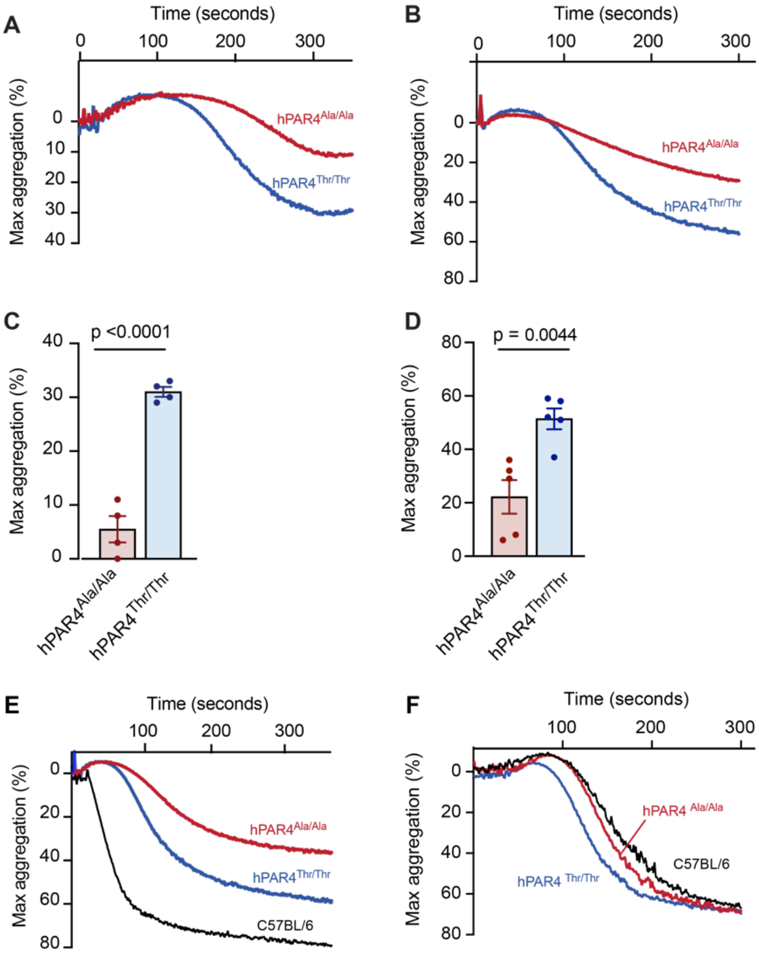

Supplemental Figure 11. Platelets from hPAR4 mice aggregate in response to mouse thrombin. Washed platelets were isolated from hPAR4$^{Ala/Ala}$ and hPAR4$^{Thr/Thr}$ mice, resuspended in Tyrode's buffer at 2x10^8 platelets/mL and stimulated with murine thrombin at the indicated concentrations. Representative tracings for thrombin at 0.25 U/mL (**A**) and 0.5 U/mL (**B**) are shown. (**C and D**) Quantification of murine thrombin-induced platelet aggregation data. N=4-5 per group. Normality was determined by a Shapiro-Wilk test and significance was determined by unpaired T-test (**C and D**). (**E and F**) Platelets from wild-type (C57BL/6) mice with mPAR4 and hPAR4$^{Ala/Ala}$ and hPAR4$^{Thr/Thr}$ mice were isolated and stimulated with 0.5 U/mL (**E**) murine thrombin to measure aggregation responses (0.5 U/mL). A non-PAR-dependent agonist, collagen (5 µg/mL), was used as a positive control (**F**). Representative tracings are shown. N=4 independent experiments.

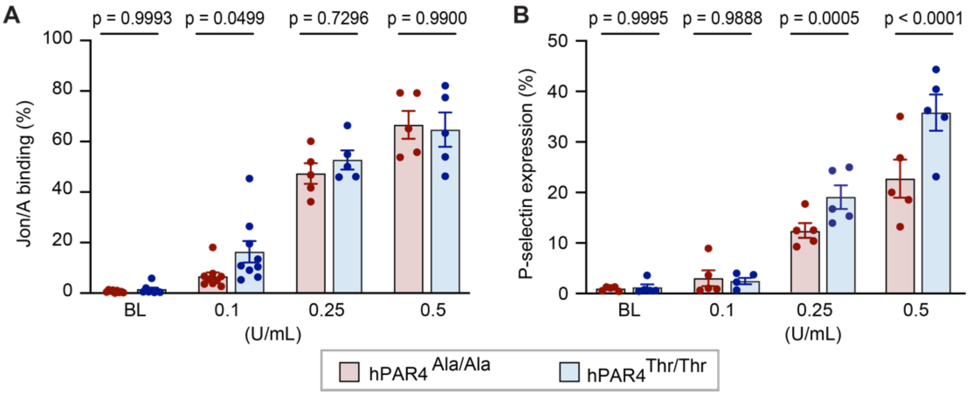

Supplemental Figure 12. Platelets from hPAR4 mice activate in response to mouse thrombin. Platelets were isolated from $hPAR4^{Ala/Ala}$ and $hPAR4^{Thr/Thr}$ mice and activated with the indicated concentrations of mouse thrombin for 15 minutes. (**A**) integrin activation (measured by JON/A binding) and (**B**) P-selectin expression was measured on CD41+ platelets by flow cytometry. N=6 per group. Significance determined by Mixed effect ANOVA with a Sidak's multiple comparisons test (**A**) and by a 2-way ANOVA with a Sidak's multiple comparisons test (**B**).

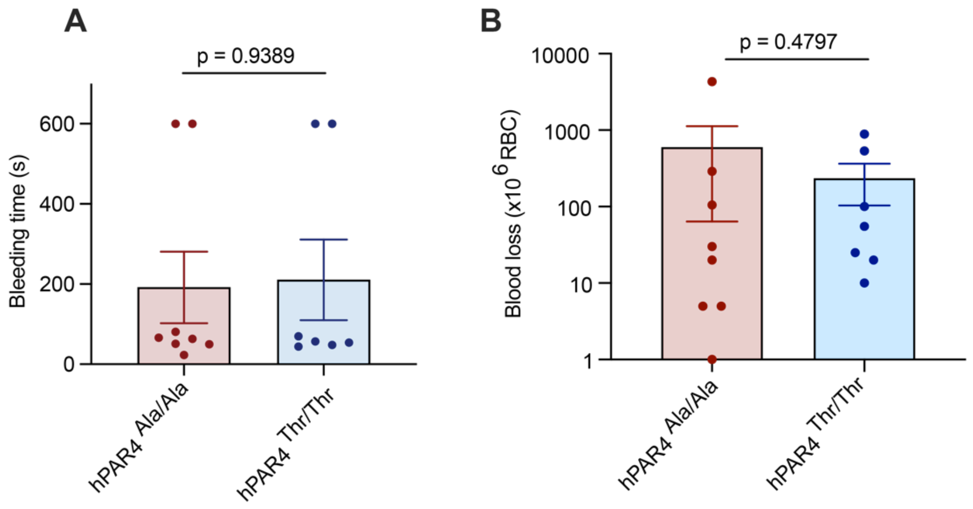

Supplemental Figure 13. Hemostasis is similar between hPAR4$^{Ala/Ala}$ and hPAR4$^{Thr/Thr}$ mice. Anesthetized hPAR4$^{Ala/Ala}$ and hPAR4$^{Thr/Thr}$ mice had the distal 4 mm of tail transected, and tails were placed in warm saline. (**A**) The time to first bleeding cessation was measured. (**B)** Total blood loss was measured by centrifuging the red blood cells lost into the saline vial and quantifying red blood cells using a Hemavet. N=7-8 per group. Normality was determined by a Shapiro-Wilk test and significance was determined by a Mann-Whitney test.

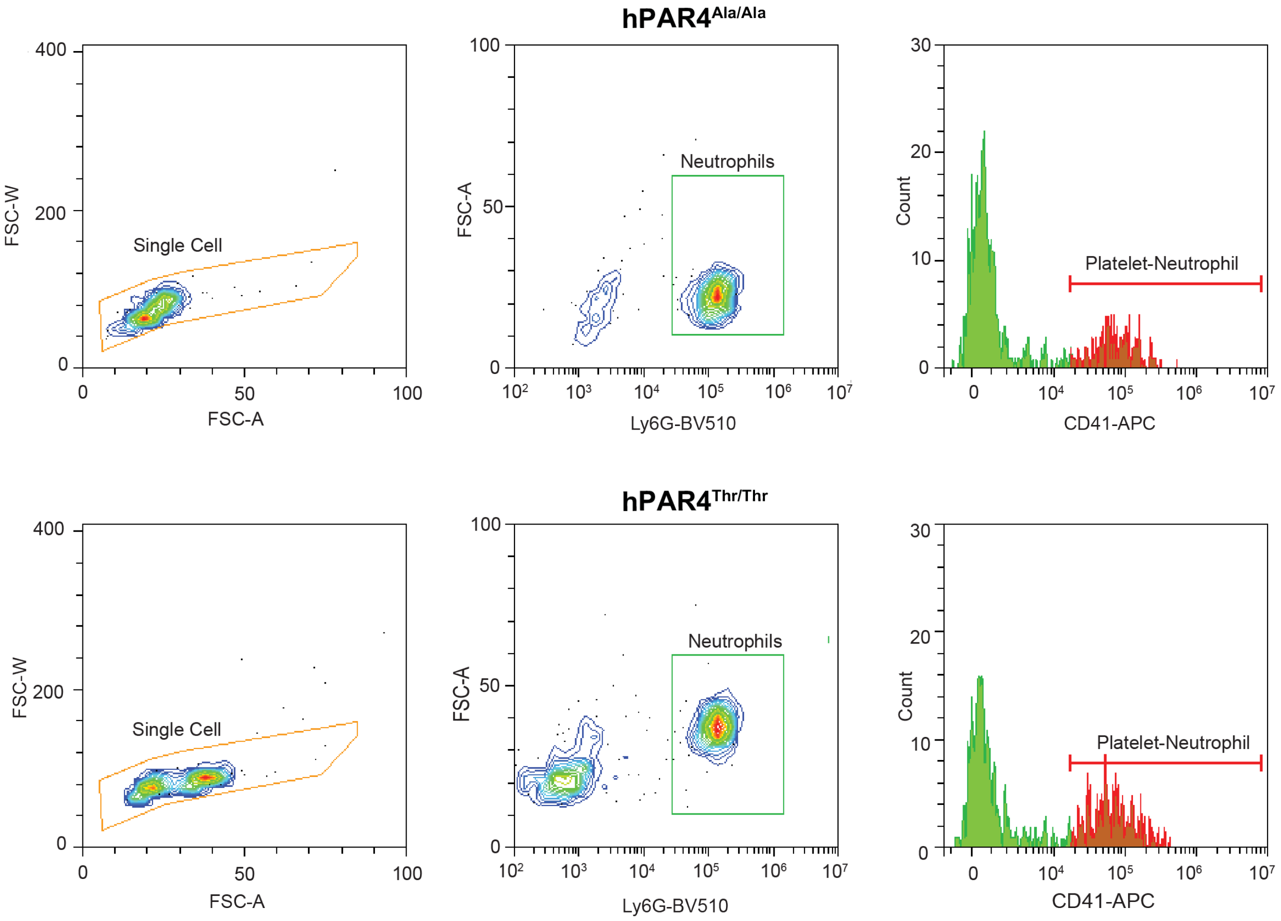

Supplemental Figure 14. Gating strategy to measure platelet-neutrophil aggregates in whole blood in humanized PAR4$^{Ala/Ala}$ and PAR4$^{Thr/Thr}$ mice after stroke. Platelet-neutrophil aggregates in hPAR4$^{Ala/Ala}$ and hPAR4$^{Thr/Thr}$ were measured in whole blood after tMCAO induced stroke by gating on Ly6G positive cells followed CD41. A minimum of 1000 Ly6G positive cells were counted.

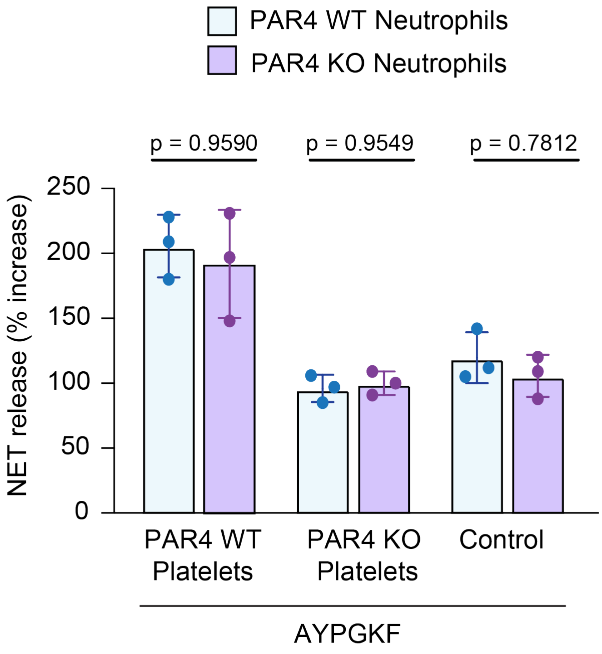

Supplemental Figure 15. PAR4-dependent platelet activation is necessary for NET induction. Platelets and neutrophils were isolated from wild-type (WT) PAR4 mice and PAR4 knockout (KO) mice. WT or PAR4 KO platelets were stimulated with AYPGKF or vehicle for 2.5 hours in the presence of the indicated neutrophil and NETs were quantified using an MPO-DNA ELISA. In some assays, WT or PAR4 KO neutrophils (Control) were stimulated with AYPGKF or vehicle for 2.5 hours in the absence of platelets and NETs were quantified using an MPO-DNA ELISA. N=3 per group. Significance was determined by 2-way ANOVA with a Sidak's Multiple Comparison test.

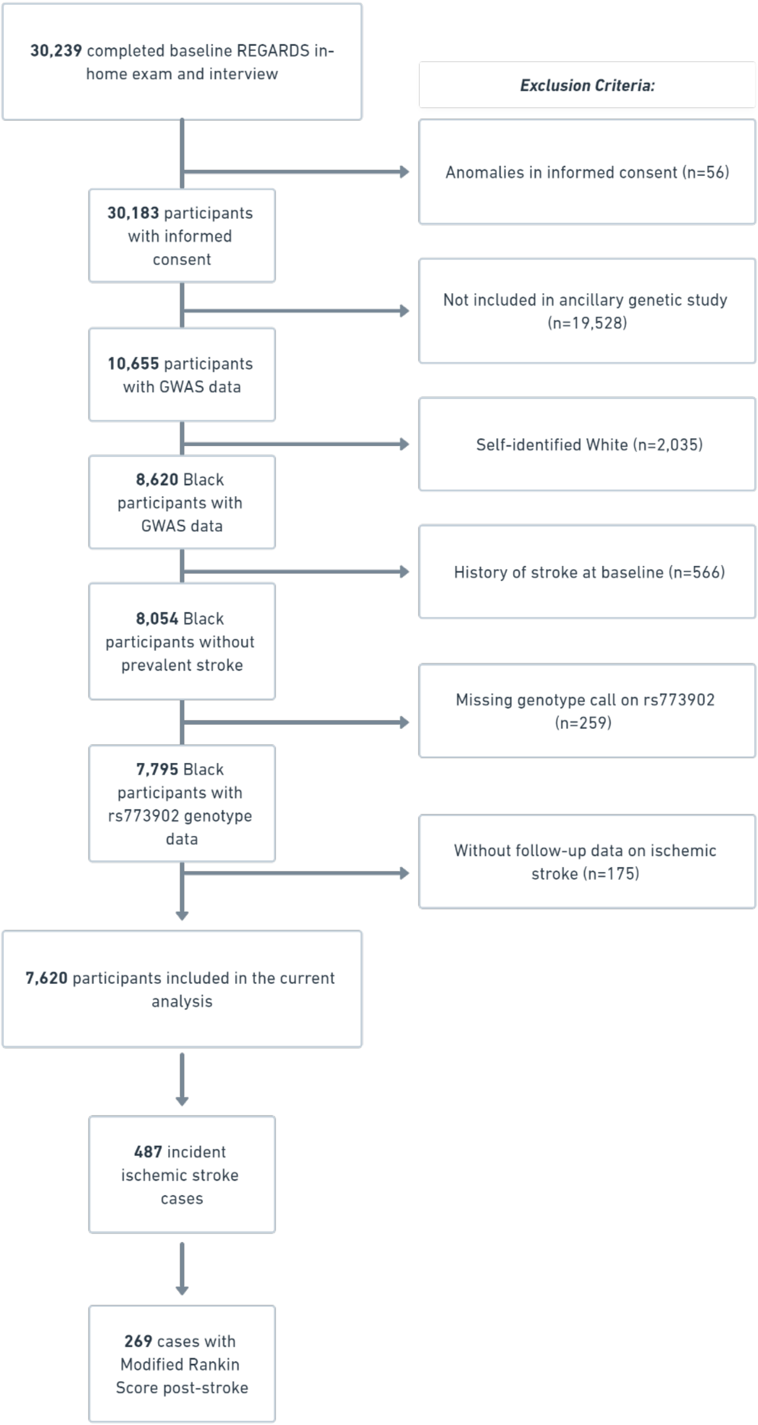

Supplemental Figure 16. Study flow chart for inclusion and exclusion of REGARDS participants for the current analysis. Excluded groups on the right are mutually exclusive.

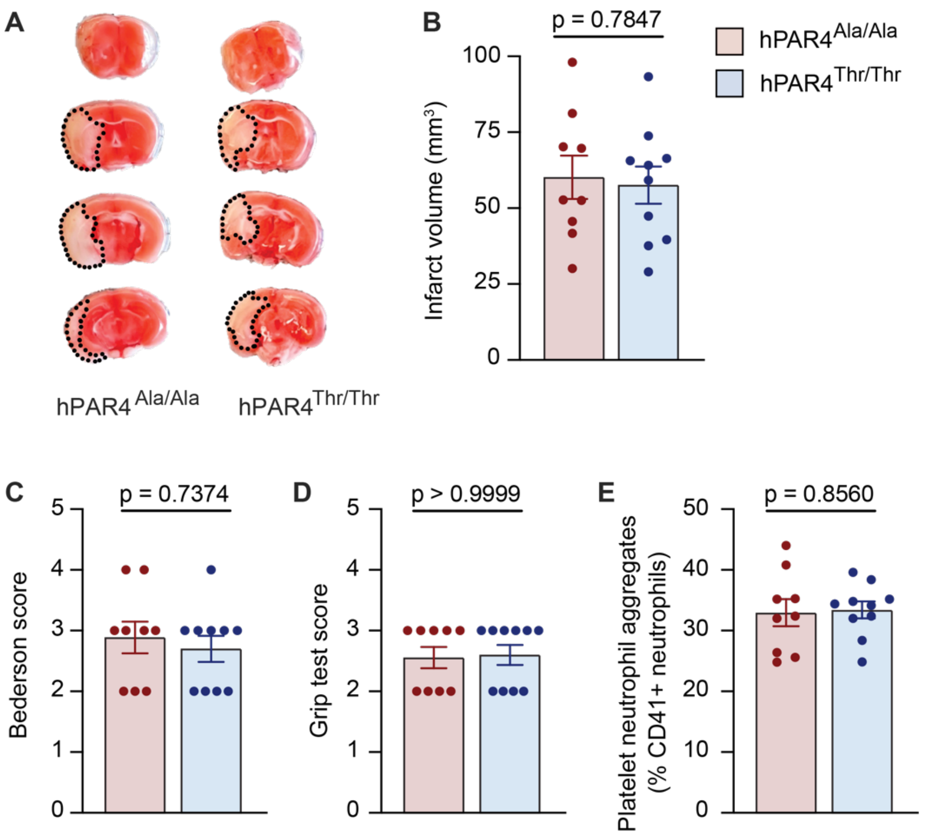

Supplemental Figure 17. Humanized PAR4$^{Ala/Ala}$ and PAR4$^{Thr/Thr}$ mice have similar stroke outcomes in severe tMCAO model. Mice were then subjected 60 minutes of tMCAO followed by 23 hours of reperfusion (severe). (**A-B**) Brains were analyzed for ischemic stroke brain damage by TTC staining, 24 hours after stroke onset. Upon TTC staining, live brain tissue will stain red, while dead brain tissue will remain white (outlined with black dotted line). Quantification of brain infarct volumes 24 hours after stroke. Images are representative of N=9-10 per group. Normality was determined by Shapiro-Wilk test while significance was determined by unpaired T-test. (**C**) The Bederson test was used to assess neurological outcome 24 hours after stroke. Normality was determined by Shapiro-Wilk test while significance was determined by Mann-Whitney U test. N=9-10 per group. (**D**) Twenty-four hours after stroke, motor function was measured using the grip test. N=9-10 per group. Normality was determined by Shapiro-Wilk test while significance was determined by Mann-Whitney U test. (**E**) Platelet-neutrophil aggregates were measured in whole blood 24 hours after stroke. N=9-10 per group. Normality was determined by Shapiro-Wilk test while significance was determined by unpaired T-test.

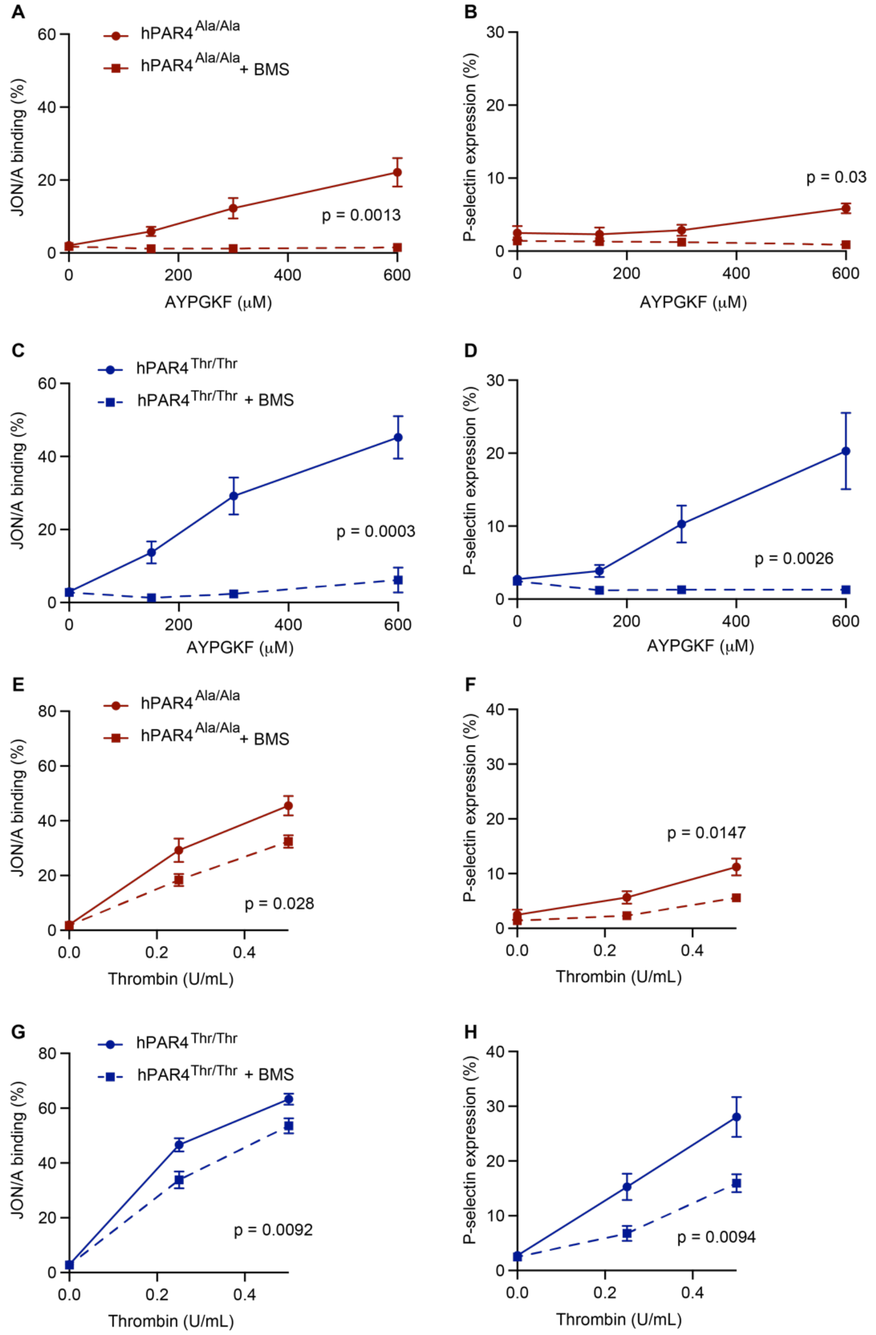

Supplemental Figure 18. BMS-986120 attenuates PAR-4 dependent platelet activation in hPAR4$^{Ala/Ala}$ and PAR4$^{Thr/Thr}$ mice. Vehicle (solid line) or BMS-986120 (10 mg/kg, dotted line) was administered i.v. to hPAR4$^{Ala/Ala}$ (red) and hPAR4$^{Thr/Thr}$ (blue) mice 1 hour before platelet isolation. Washed platelets were isolated, resuspended in Tyrode's buffer and activated with AYPGKF (**A-D**) or thrombin (**E-H**). Integrin activation was assessed by JON/A binding (**A, C, E, G**) and granule secretion was assessed by surface P-selectin expression (**B, D, F, H**). N=5-6 per group. Significance was determined by 2-way ANOVA with a Sidak's Multiple Comparison test.

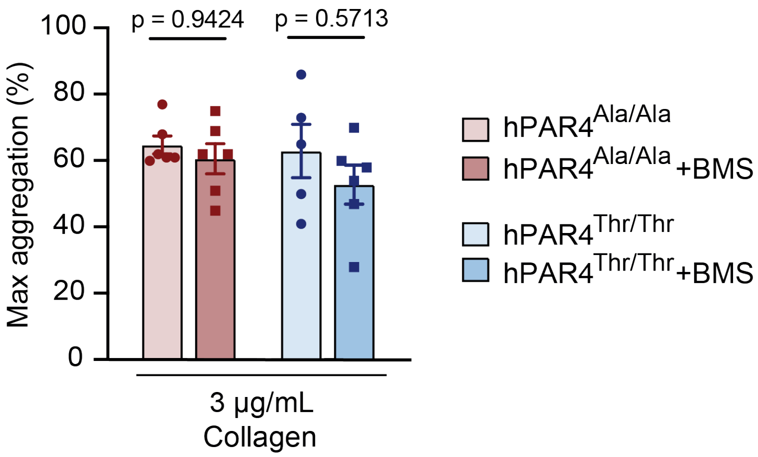

Supplemental Figure 19. BMS-986120 does not alter collagen-induced aggregation in humanized PAR4 mice. BMS-986120 (10 mg/kg) was administered i.v. to hPAR4$^{Ala/Ala}$ and hPAR4$^{Thr/Thr}$ mice 1 hour before blood was obtained. Washed platelets were resuspended in Tyrode's buffer and collagen-induced aggregation was measured. N=5-6 per group. Normality was determined by Shapiro-Wilk test while significance was determined by Kruskal-Wallis test with a Dunn's Multiple Comparisons test.

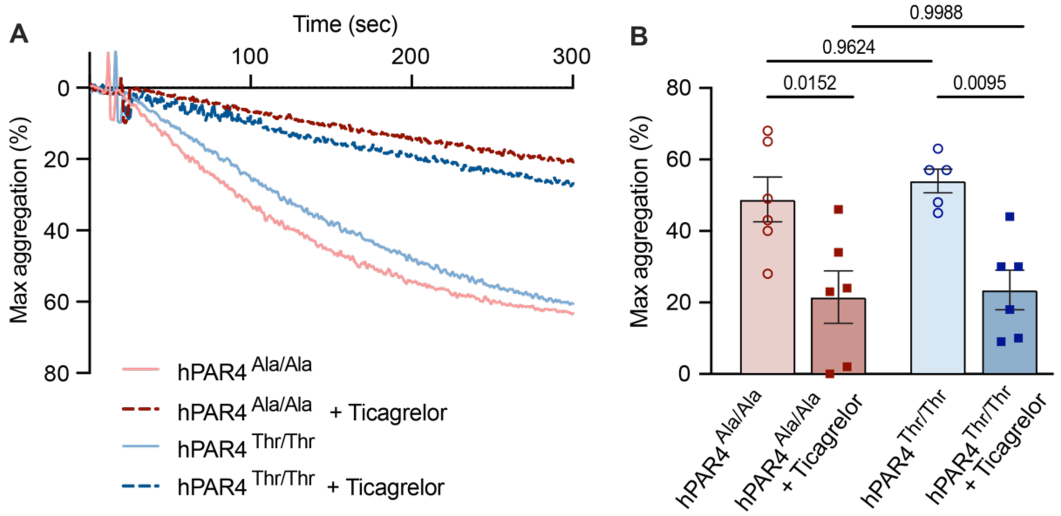

Supplemental Figure 20. Ticagrelor blocks 2MesADP-dependent platelet aggregation in humanized PAR4 mice. **(A-B)** Ticagrelor or vehicle was administered by gavage to hPAR4$^{Ala/Ala}$ and hPAR4$^{Thr/Thr}$ mice 2 hour before platelets were isolated. Washed platelets were resuspended in Tyrode's buffer and aggregation in response to 2MeSADP was measured. (**A**) A representative tracing is found in panel from N=5-6 per group. (**B)** Quantitation of aggregation tracings. N=5-6 per group. Significance determined by Mixed effect ANOVA with a Sidak's multiple comparisons test.

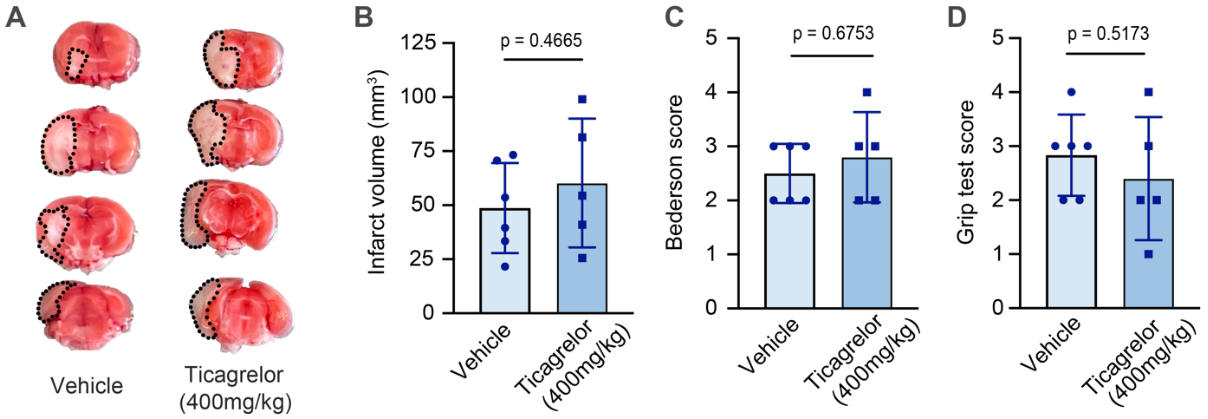

Supplemental Figure 21. High dose ticagrelor does not improve ischemic stroke outcomes in hPAR4$^{Thr/Thr}$ mice. Ticagrelor (400 mg/kg) or vehicle was administered by gavage to hPAR4$^{Thr/Thr}$ mice 2 hours before tMCAO. Mice were then subjected 60 minutes of tMCAO followed by 23 hours of reperfusion (severe). (**A**) Brain infarct volumes were quantified by staining with TTC, which colors live brain tissue red, while dead brain tissue remains white (outlined with black dotted line). Images are representative of N=5-6 per group. (**B**) Quantification of brain infarct volumes. Neurological and motor function were assessed by the Bederson (**C**) and Grip (**D**) tests, respectively. N=5-6 per group. Normality was determined by Shapiro-Wilk test while significance was determined by an unpaired T-test (**B**) and Mann-Whitney test (**C and D**).

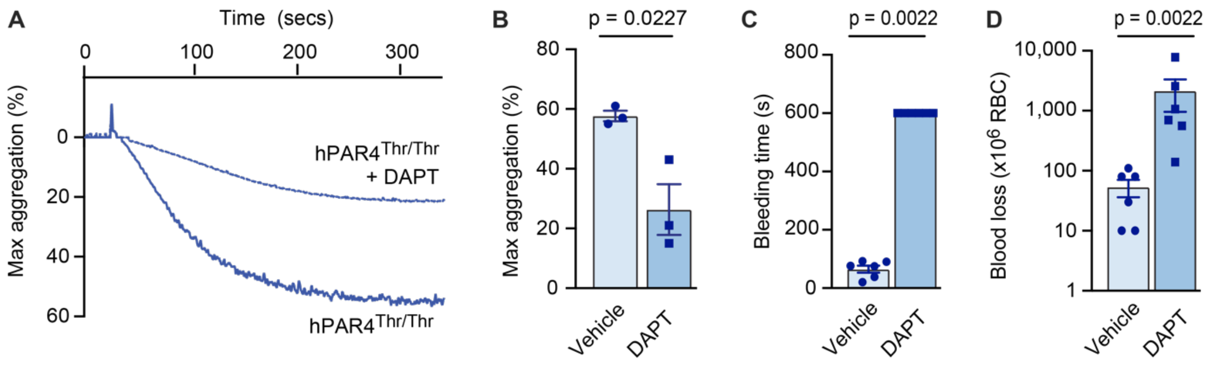

Supplemental Figure 22. DAPT alters arachidonic acid induced aggregation and hemostasis. (**A-B**) Aspirin, Ticagrelor or vehicle were administered by gavage to hPAR4$^{Thr/Thr}$ mice 2 hours before platelets were isolated. Washed platelets were resuspended in Tyrode's buffer and aggregation in response to arachidonic acid (40 μg/mL) was measured. A representative tracing is shown in panel **A**. N=3 per group. **(C-D**) Anesthetized vehicle or DAPT-treated hPAR4$^{Thr/Thr}$ mice had the distal 4 mm of tail transected, and tails were placed in warm saline. (**C**) The first time to bleeding cessation was measured. (**D)** Total blood loss was measured by centrifuging the red blood cells lost into the saline vial and quantifying red blood cells using a Hemavet. N=6 per group. Normality was determined by Shapiro-Wilk test while significance was determined by an unpaired T-test (**B**) and Mann-Whitney test (**C and D**).

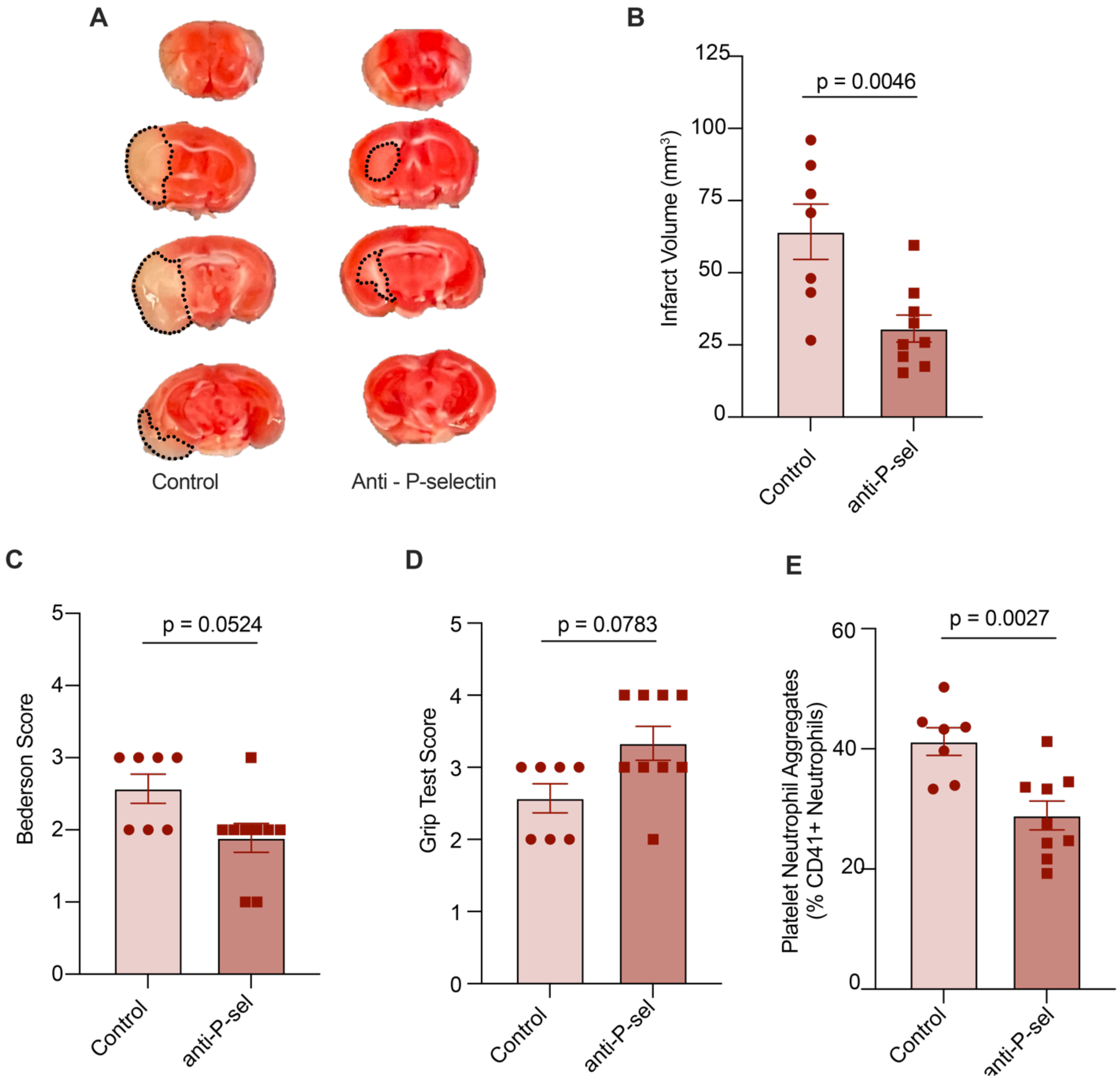

Supplemental Fig. 23. P-selectin blockade improves ischemic stroke outcomes in hPAR4$^{Ala/Ala}$ mice. Anti-P-selectin or control IgG (2 mg/kg) was administered i.v. to hPAR4$^{Ala/Ala}$ mice 1 hour before tMCAO. Mice were then subjected 60 minutes of tMCAO followed by 23 hours of reperfusion (severe). (**A**) Brain tissue was stained with TTC. Dead brain tissue is outlined with a black dotted line. Images are representative of N=7-9 per group. (**B**) Quantification of brain infarct volumes. Neurological and motor function were assessed by the Bederson (**C**) and Grip (**D**) tests. (**E**) Platelet-neutrophil aggregates were measured by flow cytometry by gating on Ly6G$^+$ neutrophils that were positive for CD41$^+$ platelets. Normality was determined by Shapiro-Wilk test while significance was determined by an unpaired T-test (**B and E**) and Mann-Whitney test (**C and D**). N=7-9 per group

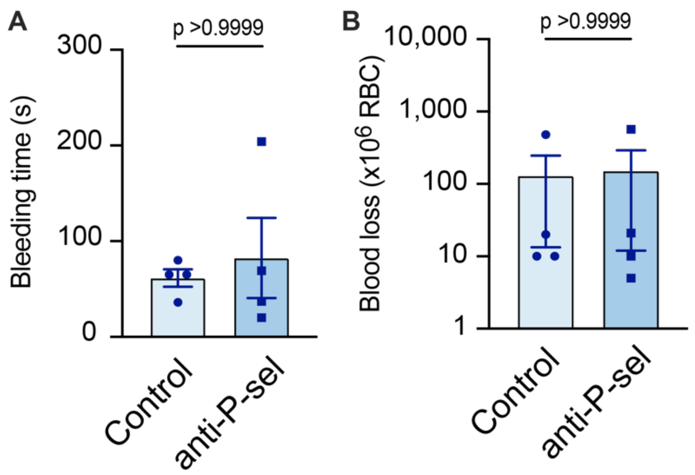

Supplemental Figure 24. Anti-P-selectin does not change hemostasis. (A-B) Tail bleeding was measured in control or anti-P-selectin treated hPAR4$^{Thr/Thr}$ mice. (**A**) The first time to bleeding cessation was measured. (**B)** Total blood loss was measured. N=4 per group. Normality was determined by Shapiro-Wilk test while significance was determined by a Mann-Whitney test (**A and B**).

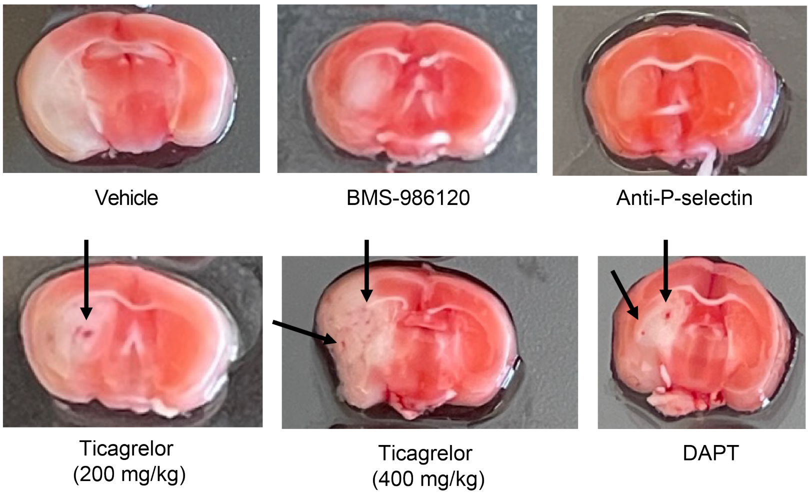

Supplemental Figure 25. Cerebral hemorrhaging associated with anti-platelet agents in hPAR4$^{Thr/Thr}$ mice. Representative images of TTC brain stains of mice treated with ticagrelor or DAPT after tMCAO. White areas indicate necrotic brain tissue, arrows indicate hemorrhages. Please note, that in parallel to bleeding complications, mice regularly develop large ischemic strokes. Images are representative of N=6-11 per treatment group.

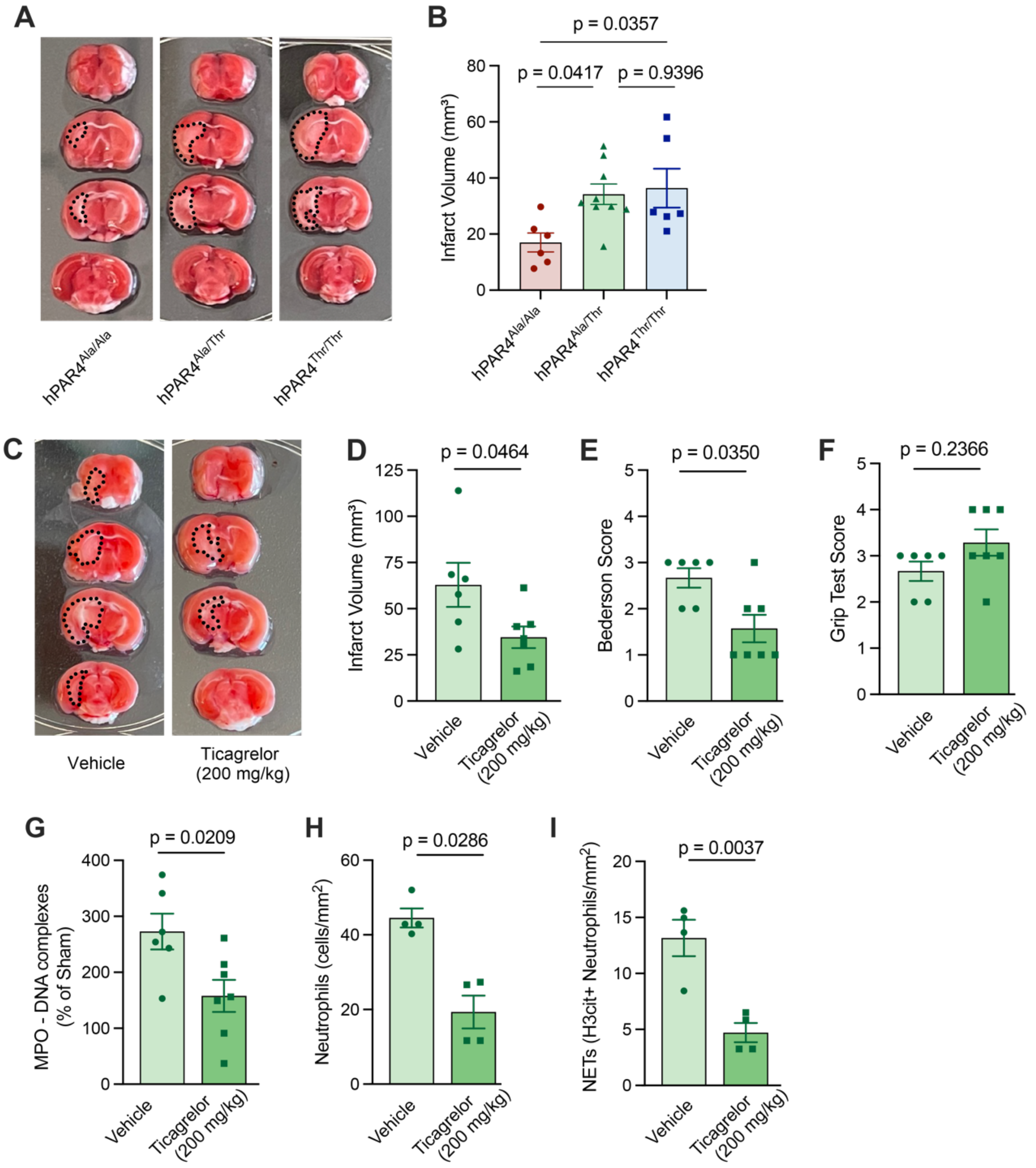

Fig. 26. Heterozygous hPAR4$^{Ala/Thr}$ mice have immediate phenotype between hPAR4$^{Ala/Ala}$ and hPAR4$^{Thr/Thr}$ mice depending on stroke severity and anti-platelet therapy. (A-B) Mice were subjected 40 minutes of tMCAO followed by 23 hours of reperfusion (moderate). Brains were analyzed for ischemic stroke brain damage by TTC staining, 24 hours after stroke onset. Upon TTC staining, live brain tissue will stain red, while dead brain tissue will remain white (outlined with black dotted line). (**A**) Images are representative of N=6-9 per group. (**B**) Quantification of brain infarct volumes 24 hours after stroke. Significance was determined with one-way ANOVA with a Tukey's Multiple Comparison test. N=6-9 per group. (**C-I**) Ticagrelor (200 mg/kg) or vehicle was administered by gavage to hPAR4$^{Ala/Thr}$ mice 2 hours before tMCAO. Mice were then subjected 60 minutes of tMCAO followed by 23 hours

of reperfusion (severe) (**C**) Brain infarct volumes were quantified by staining with TTC, which colors live brain tissue red, while dead brain tissue remains white (outlined with black dotted line). Images are representative of N=6-7 per group. (**D**) Quantification of brain infarct volumes. Neurological and motor function were assessed by the Bederson (**E**) and Grip (**F**) tests. (**G**) NET formation in the plasma was measured using an MPO-DNA ELISA. (**H**) Neutrophils were identified by myeloperoxidase (MPO) and DNA (DAPI) statins and presented as neutrophils per area of infarcted brain tissue. (**I**) Brain tissue was stained for myeloperoxidase (MPO), citrullinated histone H3 (H3cit) and DNA (DAPI) and NET-forming neutrophils were quantified as the percentage of MPO positive cells that were also H3cit positive. Normality was determined by Shapiro-Wilk test while significance was determined by a unpaired test or Mann-Whitney test. N=6-7 per group

References

1. Armstrong ND, Srinivasasainagendra V, Patki A, Tanner RM, Hidalgo BA, Tiwari HK, et al. Genetic Contributors of Incident Stroke in 10,700 African Americans With Hypertension: A Meta-Analysis From the Genetics of Hypertension Associated Treatments and Reasons for Geographic and Racial Differences in Stroke Studies. *Front Genet.* 2021;12:781451.
2. Das S, Forer L, Schonherr S, Sidore C, Locke AE, Kwong A, et al. Next-generation genotype imputation service and methods. *Nat Genet.* 2016;48(10):1284-7.
3. Price AL, Patterson NJ, Plenge RM, Weinblatt ME, Shadick NA, and Reich D. Principal components analysis corrects for stratification in genome-wide association studies. *Nat Genet.* 2006;38(8):904-9.
4. Martin M. Cutadapt removes adapter sequences from high-throughput sequencing reads. *EMBnetjournal.* 2011;17(1):10-2.
5. Li H, and Durbin R. Fast and accurate short read alignment with Burrows-Wheeler transform. *Bioinformatics.* 2009;25(14):1754-60.
6. Danecek P, Bonfield JK, Liddle J, Marshall J, Ohan V, Pollard MO, et al. Twelve years of SAMtools and BCFtools. *Gigascience.* 2021;10(2).
7. Ewels P, Magnusson M, Lundin S, and Kaller M. MultiQC: summarize analysis results for multiple tools and samples in a single report. *Bioinformatics.* 2016;32(19):3047-8.
8. Thorvaldsdottir H, Robinson JT, and Mesirov JP. Integrative Genomics Viewer (IGV): high-performance genomics data visualization and exploration. *Brief Bioinform.* 2013;14(2):178-92.
9. Shen W, Le S, Li Y, and Hu F. SeqKit: A Cross-Platform and Ultrafast Toolkit for FASTA/Q File Manipulation. *PLoS One.* 2016;11(10):e0163962.
10. Kent WJ. BLAT--the BLAST-like alignment tool. *Genome Res.* 2002;12(4):656-64.
11. Middleton EA, Rowley JW, Campbell RA, Grissom CK, Brown SM, Beesley SJ, et al. Sepsis alters the transcriptional and translational landscape of human and murine platelets. *Blood.* 2019;134(12):911-23.
12. Renna SA, Michael JV, Kong X, Ma L, Ma P, Nieman MT, et al. Human and mouse PAR4 are functionally distinct receptors: Studies in novel humanized mice. *J Thromb Haemost.* 2022;20(5):1236-47.
13. Mao Y, Zhang M, Tuma RF, and Kunapuli SP. Deficiency of PAR4 attenuates cerebral ischemia/reperfusion injury in mice. *J Cereb Blood Flow Metab.* 2010;30(5):1044-52.
14. Portier I, Vanhoorelbeke K, Verhenne S, Pareyn I, Vandeputte N, Deckmyn H, et al. High and long-term von Willebrand factor expression after Sleeping Beauty transposon-mediated gene therapy in a mouse model of severe von Willebrand disease. *J Thromb Haemost.* 2018;16(3):592-604.
15. Manne BK, Campbell RA, Bhatlekar S, Ajanel A, Denorme F, Portier I, et al. MAPK-interacting kinase 1 (Mnk1) regulates platelet production, activation, and thrombosis. *Blood.* 2022.
16. Denorme F, Portier I, Kosaka Y, and Campbell RA. Hyperglycemia exacerbates ischemic stroke outcome independent of platelet glucose uptake. *J Thromb Haemost.* 2021;19(2):536-46.
17. Denorme F, Manne BK, Portier I, Eustes AS, Kosaka Y, Kile BT, et al. Platelet necrosis mediates ischemic stroke outcome in mice. *Blood.* 2020;135(6):429-40.
18. Denorme F, Portier I, Rustad JL, Cody MJ, de Araujo CV, Hoki C, et al. Neutrophil extracellular traps regulate ischemic stroke brain injury. *J Clin Invest.* 2022;132(10).
19. Campbell RA, Vieira-de-Abreu A, Rowley JW, Franks ZG, Manne BK, Rondina MT, et al. Clots Are Potent Triggers of Inflammatory Cell Gene Expression: Indications for Timely Fibrinolysis. *Arterioscler Thromb Vasc Biol.* 2017;37(10):1819-27.
20. Middleton EA, He XY, Denorme F, Campbell RA, Ng D, Salvatore SP, et al. Neutrophil extracellular traps contribute to immunothrombosis in COVID-19 acute respiratory distress syndrome. *Blood.* 2020;136(10):1169-79.

21. Campbell RA, Campbell HD, Bircher JS, de Araujo CV, Denorme F, Crandell JL, et al. Placental HTRA1 cleaves alpha1-antitrypsin to generate a NET-inhibitory peptide. *Blood.* 2021;138(11):977-88.
22. Whitley MJ, Henke DM, Ghazi A, Nieman M, Stoller M, Simon LM, et al. The protease-activated receptor 4 Ala120Thr variant alters platelet responsiveness to low-dose thrombin and protease-activated receptor 4 desensitization, and is blocked by non-competitive P2Y12 inhibition. *J Thromb Haemost.* 2018;16(12):2501-14.
